# Evaluating precision medicine approaches for gene therapy in patient-specific cellular models of Bietti crystalline dystrophy

**DOI:** 10.1172/jci.insight.177231

**Published:** 2024-07-16

**Authors:** Yao Li, Richard R. Yang, Yong-Shi Li, Chun-Wei Hsu, Laura A. Jenny, Yang Kong, Merry Z.C. Ruan, Janet R. Sparrow, Stephen H. Tsang

**Affiliations:** 1 Jonas Children’s Vision Care, Department of Ophthalmology, Columbia University, New York, New York, USA.; 2 Reflection Biotechnologies, New Territories, Hong Kong.; 3 Vagelos College of Physicians and Surgeons, Columbia University, New York, New York, USA.; 4 Department of Pathology and Cell Biology, Columbia University Irving Medical Center, New York, New York, USA.; 5 Edward S. Harkness Eye Institute, New York-Presbyterian Hospital, New York, New York, USA.; 6 Columbia Stem Cell Initiative, Columbia University, New York, New York, USA.

**Keywords:** Ophthalmology, Therapeutics, Gene therapy, Genetic diseases, iPS cells

## Abstract

Patient-specific induced pluripotent stem cell–derived (iPSC-derived) cell lines allow for therapies to be tailored to individual patients, increasing therapeutic precision and efficiency. Bietti crystalline dystrophy (BCD) is a rare blinding disease estimated to affect about 67,000 individuals worldwide. Here, we used iPSC-derived retinal pigment epithelium (iRPE) cells from patients with BCD to evaluate adeno-associated virus–mediated (AAV-mediated) gene augmentation therapy strategies. We found that BCD iRPE cells were vulnerable to blue light–induced oxidative stress and that cellular phenotype can be quantified using 3 robust biomarkers: reactive oxygen species (ROS), 4-hydroxy 2-nonenal (4-HNE) levels, and cell death rate. Additionally, we demonstrated that AAV-mediated gene therapy can significantly reduce light-induced cell death in BCD iRPE cells. This is the first proof-of-concept study to our knowledge to show that AAV-*CYP4V2* gene therapy can be used to treat light-induced RPE damage in BCD. Furthermore, we observed significant variability in cellular phenotypes among iRPE from patients with BCD of divergent mutations, which outlined genotype-phenotype correlations in BCD patient–specific cell disease models. Our results reveal that patient-specific iRPE cells retained personalized responses to AAV-mediated gene therapy. Therefore, this approach can advance BCD therapy and set a precedent for precision medicine in other diseases, emphasizing the necessity for personalization in healthcare to accommodate individual diversity.

## Abstract 2

This study utilized patient cell-based disease models to screen gene therapy strategies, and found personalized preference to different treatments and distinct therapeutic efficacy.

## Introduction

Gene therapy has become a powerful tool for developing treatments for genetic disorders since its first FDA approval in 2017. Interspecies differences between animals and humans are a limitation of animal-based testing that happens in preclinical trials. Additionally, animal models cannot factor in genotype differences among patients. Importantly, individual differences such as genotypes, genetic polymorphisms, and individual cell surface receptor expression levels could have a significant effect on gene therapy transduction and treatment efficacy (1). It is essential to consider individual patient responses in gene therapy development to maximize the clinical benefits. One of the barriers to moving a clinical trial forward is the variation in response to therapy from individual patients. Precision medicine approaches are one such way to overcome this barrier, since patient-specific cell lines provide a platform to tailor personalized treatments. Divergent genotypes may cause different disease expressivity, and individual genetic features (i.e., gene modifiers and the receptor genes that affect transfection/transduction) may lead to variable treatment responses.

Patient-specific induced pluripotent stem cells (iPSCs) allow treatments to be tested on patient cells before they are enrolled into trials. In recent years, patient-specific cell lines (i.e., ex vivo tissues and organoids) have been used to model certain genetic disorders (2–4). Moreover, patient iPSCs may be used to fill the gaps in modeling diseases with no suitable animal models and generate preclinical proof-of-concept efficacy data to support Investigational New Drug (IND) application for clinical trials (5). Our results provide an example of using patient-specific iPSC-based cellular models to screen for individualized optimal vectors and dosing, which can be referenced in precision gene therapy. Furthermore, our results suggest that adeno-associated virus–mediated (AAV-mediated) gene therapy human clinical trials might benefit from testing on patient-derived cells to prioritize inclusion into gene supplementation Phase II trials.

Bietti crystalline dystrophy (BCD), also known as Bietti crystalline corneoretinal dystrophy (OMIM 210370), is an autosomal recessive retinal degenerative disorder caused by mutations in the *CYP4V2* gene (6). Clinically, BCD is associated with retinal pigment epithelium (RPE) cell death (7), and the onset age and phenotype manifestations of patients with BCD are highly variable (7). Genetically, more than 100 *CYP4V2* mutations have been reported (8). Given the wide genotype and phenotype variability among patients, BCD is an ideal disease for studying patient individual differences. Moreover, an in-depth worldwide *CYP4V2* mutation carrier frequency and BCD genetic prevalence study estimated that BCD may affect about 67,000 individuals worldwide and revealed that the most common *CYP4V2* mutations are different among various populations (9). These factors underpin the importance of establishing BCD patient–specific iPSC-based models of different ethnic backgrounds with distinct mutations to generate highly representative results and assess individual variations. In recent years, researchers began to use iPSC-derived RPE (iRPE) cells to study BCD and to test drug candidates, including gene therapies (10–12). However, these studies did not research individual patient differences due to the limited number of patient samples and mutations. Furthermore, no study to date, to our knowledge, has tested different AAV serotypes or doses.

In this study, we established iRPE cell lines from 6 patients with BCD with 3 different ethnic origins harboring distinct *CYP4V2* mutations. Interestingly, these iRPE cell lines exhibited individual differences in phenotypes, including the clinically significant phenotype of RPE cell death. Importantly, our iRPE from patients with BCD successfully overcame the limitations observed in *Cyp4v3*^–/–^ mouse models (*Cyp4v3* is the murine ortholog of human *CYP4V2*) (13–16). Our iRPE cultures mimic cell death seen in patients with BCD. It should be noted that no RPE cell death has been reported in the *Cyp4v3*^–/–^ mouse model (13–15). Due to the absence of appropriate *Cyp4v3*^–/–^ mouse models, the mechanisms underlying RPE death in BCD remain unclear.

In addition, we tested different AAV-*CYP4V2* vector serotypes and dosages in these iRPE cell lines. Our results reveal that the AAV vectors achieved different treatment efficacy in different patients’ iRPE cell lines. These results explain the variability at the cellular level in gene therapy efficacy among patients and show the ability of patient cell–based “disease-on-dish” can contribute to developing precision medicine approaches in future gene therapies.

## Results

### Building a BCD cellular disease platform with a diverse pool of BCD patient–specific iRPE cell lines.

**Figure 1:**
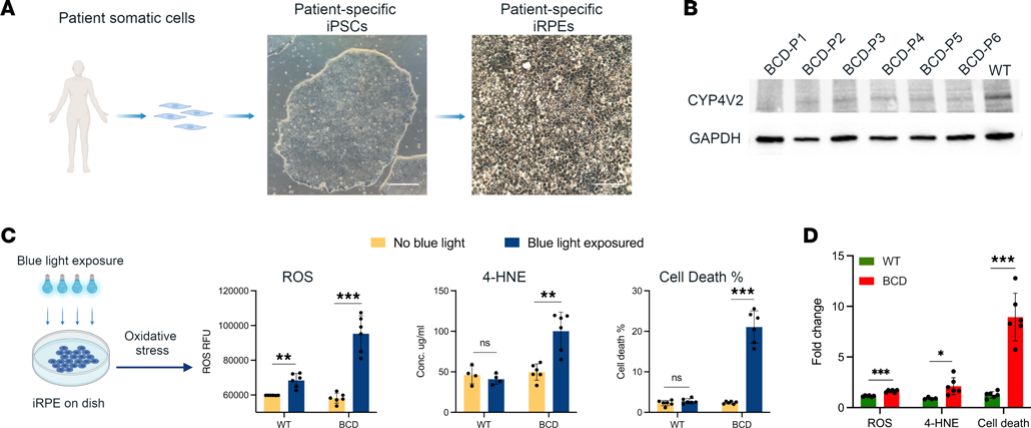
BCD iRPE cells are susceptible to shortwave light–induced (blue light–induced) oxidative stress, and this phenotype can be quantified by monitoring ROS, 4-HNE and cell death rate. (**A**) Schematic showing the establishment of the patient-specific iPSC-derived cell-based BCD disease platform. Scale bar: 50 μm. (**B**) Immunoblot showing CYP4V2 expression levels are lower in iRPE cells from all 6 patients with BCD compared with iRPE cells from WT donors. GAPDH serves as a loading control. (**C**) Scheme of blue light induced oxidative stress application: 430 nm blue light exposure. Quantification chart of ROS, 4-HNE level, and cell death rate of iRPE from WT donors and patients with BCD. (**D**) Relative fold change of ROS, 4-HNE level, and cell death rate in WT and BCD iRPE cells. Data are presented as mean ± SD, *n* = 6 biological replicates in all groups, except *n* = 4 biological replicates in WT group of 4-HNE. **P < 0.01, ***P < 0.001.

In this study, we built a pool of iPSC lines from 6 unrelated patients with BCD of 3 ethnicities with distinct *CYP4V2* mutations (referred to as BCD-P1 to BCD-P6) and differentiated them into iRPE cells (Figure 1A). These cell lines harbor the common *CYP4V2* mutations found in East Asian (c.802–8_810del17insGC, c.992A > C and c.1091–2A > G) (9, 17, 18), European (c.1198C > T and c.332T > C) (9, 17, 19), and South Asian populations of patients with BCD (c.197T > G) (7, 9). These patient cell lines contain both homozygous (BCD-P1, -P4, -P5, and -P6) and compound heterozygous (BCD-P2 and -P3) *CYP4V2* mutations with a total of 7 different mutations affecting 6 of 11 exons of *CYP4V2* gene. The cell lines include different mutation types (indel, missense, and splice acceptor variant) (Table 1). After the patient-specific iPSCs were differentiated into RPE cell fate (Supplemental Figure 1, A–D; supplemental material available online with this article; https://doi.org/10.1172/jci.insight.177231DS1), we cultured the iRPE cells for 6–8 weeks to allow them to reach a fully mature state. We then probed the CYP4V2 expression levels in these iRPE cells. In all 6 BCD iRPE cell lines, expression levels of CYP4V2 were lower than in WT iRPE cells (Figure 1B). Besides, we have also probe CYP4V2 expression level in multiclone–derived iRPE cells from the same patient with BCD to avoid intraindividual variations, and no difference had been observed in iRPE cells derived from different clones of the same patient with BCD (Supplemental Figure 1E).

CYP4 enzymes are traditionally associated with endogenous fatty acid metabolism (20). A previous study suggested that the CYP4V2 protein may be involved in the omega-hydroxylation of polyunsaturated fatty acids (PUFAs) and that mutant CYP4V2 may result in abnormal accumulation of omega-3 and omega-6 PUFAs such as docosahexaenoic acid (DHA) and arachidonic acid (AA) in HepG2 cells (21). To probe the PUFAs levels in our BCD patient–specific iRPE cells, untargeted lipidomics of free fatty acids (FFAs) was performed on multiple individual WT iRPE cell lines (iRPE cell lines from 6–8 individual healthy donors) and BCD iRPE cell lines (5–6 individual iRPE lines from 2 patients with BCD, BCD-P1 and BCD-P2, 2–3 iRPE lines from 2–3 individual iPSC clones of each patient with BCD). Nearly all tested medium-chained PUFAs were significantly higher in BCD iRPE cells (Supplemental Figure 2A) than in WT control iRPE cells. Among all probed items, the omega-6 PUFA AA was the major accumulated PUFA in BCD iRPE cells. Omega-3 and omega-6 PUFAs of 20-Carbon and 22-Carbon are significantly higher in BCD iRPE cells compared with WT.

### BCD iRPE cellular models are more vulnerable to blue light–induced oxidative stress, and in vitro disease phenotypes are quantifiable.

We sought to investigate how the accumulated PUFAs cause cell death in BCD RPE. Double bonds in PUFAs are target substrates to propagate oxidative stress (22) and will degrade into aldehydes, which are highly reactive species that participate in the cellular pathways that may lead to apoptosis. In BCD iRPE cells, omega-6 PUFA abnormally accumulates, and the major end-product of omega-6 PUFA from peroxidation was aldehyde 4-hydroxynonenal (4-HNE), which altered cell signaling and directly caused cell death (23).

Here, to recapitulate the RPE cell death phenotype and determine if the terminal product of omega-6 PUFA correlates with BCD iRPE death in vitro, we exposed BCD (6 iRPE cell lines from 6 individual patients with BCD) and WT iRPE cells (multiple iRPE cell lines from 4–6 individual healthy donors) to 430 nm wavelength blue light (referred to as “blue light” hereafter) to introduce the oxidative stress (Figure 1C). Next, we investigated reactive oxygen species (ROS), a marker for oxidative stress, and 4-HNE levels in iRPE cells from 6 patients with BCD and WT donors to determine if ROS and the terminal product of omega-6 PUFA correlate with BCD iRPE death.

After exposure to blue light, the average ROS level increased in both BCD and WT iRPE cells, indicating elevated levels of oxidative stress (Figure 1C). However, after blue light exposure, the average ROS level in BCD iRPE cells increased more than in WT iRPE cells. The relative change of average ROS fluorescence intensity, relative fluorescent units (RFU), before and after blue light exposure in WT iRPE cells, was 8,584 RFU; in BCD iRPE cells, it was 37,119 RFU. The relative ROS change in BCD iRPE cells is more than 4 times higher than in WT iRPE cells (Figure 1C).

To monitor the 4-HNE level in iRPE cells, we measured 4-HNE protein-adduct concentration by ELISA. In the WT group, the 4-HNE average concentration was stable after blue light exposure (46.19 μg/mL without blue light exposure and 40.96 μg/mL after exposure). In the BCD group, before blue light exposure, the average 4-HNE concentration was 49.75 μg/mL, similar to that of the WT group. However, after blue light exposure, average 4-HNE concentration more than doubled in BCD iRPE, reaching 100.18 μg/mL (Figure 1C).

This trend was shown also in cell viability tests. Cell viability reagents Calcein AM and propidium iodide (PI) were used to label live and dead iRPE cells to quantify iRPE cell death rates. Without blue light exposure, the average iRPE cell death rates in the WT and BCD groups were 2.30% and 2.40%, respectively. After blue light exposure, the iRPE cell death rate in the WT group remained stable at 2.76%, but in the BCD group, the cell death rate increased dramatically to 21.06% (Figure 1C), which is about 7.6 times of the average cell death rate in WT iRPE cells exposed to blue light (2.76%). The iRPE cell–based disease model of patients with BCD showed significant increases in 4-HNE concentrations and cell death rates after exposure to blue light. There was no significant difference in both outcomes before and after blue light exposure in WT iRPE cells. The relative change of ROS, 4-HNE level, and cell death rates in WT iRPE and BCD iRPE, before and after blue light exposure, are summarized in Figure 1D. Our results reveal that RPE cells from patients with BCD are highly susceptible to blue light–induced cell death.

To further validate the cellular phenotype that we found in BCD patient–specific iRPE cells, we established an isogenic iPSC line from BCD-P1 with a CRISPR/Cas9 genome editing system and successfully repaired in both alleles the homozygous 17 bp deletion mutation c.802-8_810del17insGC in the *CYP4V2* gene, the most common mutation among patients with BCD (9, 17) (Supplemental Figure 3A). We then differentiated this repaired isogenic iPSC line into the RPE cell fate (Supplemental Figure 3B). We found that BCD-P1 isogenic iRPE cells express higher levels of CYP4V2 compared with BCD-P1 parental iRPE cells (Supplemental Figure 3B). There was no significant change in ROS level, 4-HNE concentration, or cell death rate of BCD isogenic iRPE before or after blue light exposure (Supplemental Figure 3, C and D). This evidence further validated that the increase in cell death in BCD iRPE cells after blue light exposure was directly linked to *CYP4V2* mutations.

### Cellular phenotype differences can be observed among iRPE from individual patients with BCD who have divergent mutations in CYP4V2 gene.

**Figure 2:**
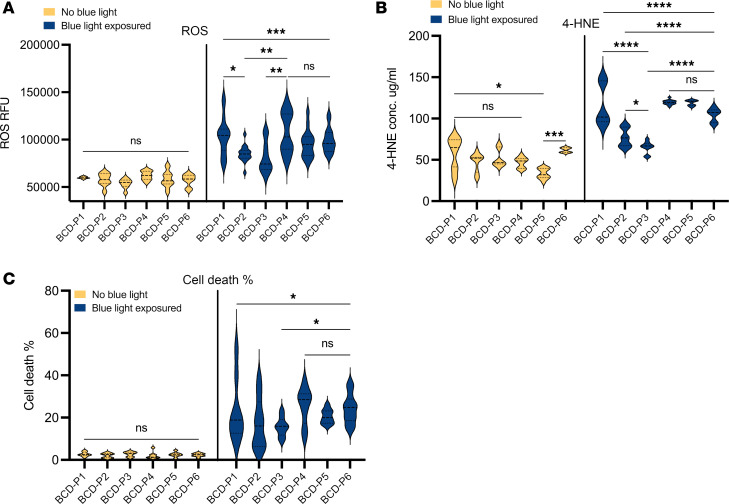
Significant cellular phenotype differences can be observed in iRPE cells of 6 individual patients with BCD with divergent mutations in *CYP4V2* gene. (**A** and **B**) Violin plot of comparison of oxidative stress induced (**A**) ROS level, (**B**) 4-HNE concentration, and (**C**) cell death rate of iRPE cells of 6 individual patients with BCD, *n* = 6–13. Significance in ROS and 4-HNE chart is calculated by 1-way ANOVA with Tukey’s multiple comparisons tests. Significance in cell death rate chart is calculated by Welch ANOVA test with multiple comparisons. *P < 0.05, **P < 0.005, ***P < 0.001, ****P < 0.0001.

During the BCD disease modeling process, we observed that the individual BCD cellular phenotype (ROS, 4-HNE level, and cell death rate) varied in BCD iRPE cells from patients of different mutations in their *CYP4V2* gene. According to these observations, we performed a statistical analysis of direct comparison of the ROS, 4-HNE level, and cell death rate among all the iRPE in 6 patients with BCD. Before blue light exposure, there was no significant difference in ROS or cell death rates among all the iRPE in 6 patients with BCD. For 4-HNE, before blue light, BCD-P5 had a significantly lower concentration of 4-HNE than the other 5 BCD iRPE samples. After blue light exposure, there were significant cellular phenotype differences among the iRPE in 6 patients with BCD in all the 3 biomarkers. We also performed multicomparison with 1-way ANOVA and observed significant phenotype differences among various patients. In brief, there were significant phenotype differences between BCD-P1 and BCD-P2, as well as between BCD-P1 and BCD-P3, in ROS levels and 4-HNE; there were also significant phenotype differences between BCD-P2 and BCD-P4 in ROS and among BCD-P2 to BCD-P6 in 4-HNE and significant phenotype differences between BCD-P3 and BCD-P4 in ROS and between BCD-P2 and BCD-P3, as well as among BCD-P3 to BCD-P6 in 4-HNE and cell death rate. In addition, there was no significant phenotype difference among BCD-P4, BCD-P5, and BCD-P6 in all 3 tested phenotype biomarkers (Figure 2).

### AAV serotypes screening to discover optimal vectors for clinical trials.

We performed a preliminary screen to test multiple AAV vectors of different serotypes (vector details summarized in Supplemental Table 1) on 5–6 iRPE cell lines from BCD-P1 and BCD-P2 (2–3 iRPE lines from 2–3 individual iPSC clones of each patient with BCD). We also used multiple individual WT iRPE cell lines as controls to measure the AAV treatment efficacy. The accumulated PUFA levels in these AAV-treated BCD iRPE cells were examined. FFA lipidomic results show that all the tested AAV-*CYP4V2* vectors reduced the average level of major PUFAs (e.g., AA and DHA) that had accumulated in BCD iRPE cells (Supplemental Figure 2B). Overall, AAV2 and AAV5 decreased abnormal PUFA levels the most in BCD iRPE.

### Application of optimal AAV vectors with different dosages to BCD patient–specific cell-based disease model platforms and patient-specific cell model elucidates individual differences in gene therapy treatment efficacy.

**Figure 3:**
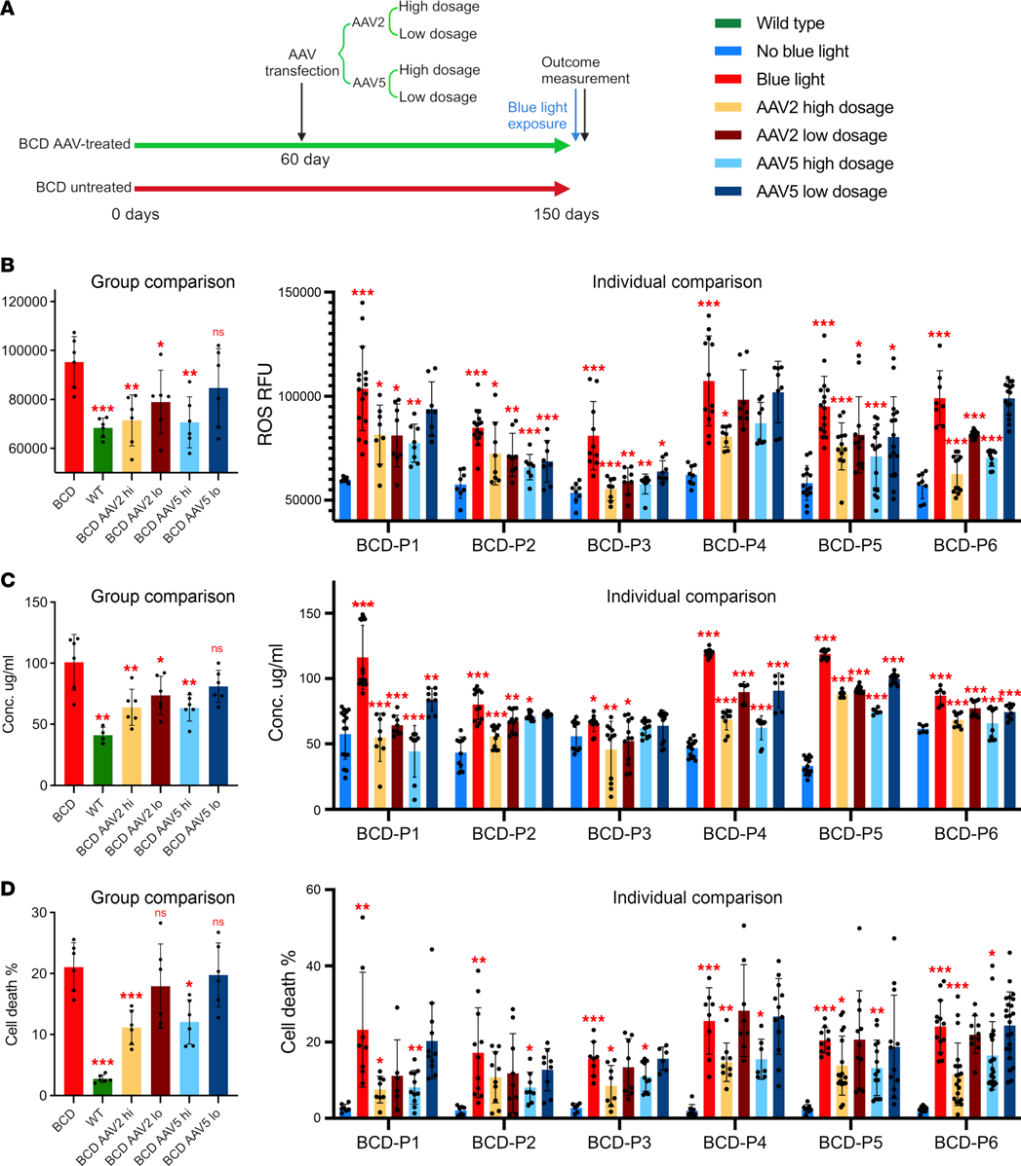
Personalized preference to different AAV treatment strategies and distinct outcome measurements of therapeutic efficacy from iRPE in individual patients with BCD. (**A**) Schematic of AAV gene augmentation therapy strategies, which indicates the timing and dosage of the AAV application. High dosage equals AAV MOI of 1 × 10^5^ (vg/cell) and low dosage equals AAV MOI of 1 × 10^4^ (vg/cell). (**B**–**D**) Comparison of AAV therapeutic efficacy from different vectors and dosages (indicated by color legend) as measured by ROS levels (**B**), 4-HNE concentration (**C**), and cell death rate under AAV treatment plan (**D**) according to **A**, respectively. In **B**, **C**, and **D**, respectively, left charts present the outcome comparison of iRPE from grouped patients with BCD of each AAV treatment strategy. Right charts present outcome measurement comparison of iRPE from individual patients with BCD of each AAV treatment strategy. All data are presented as mean ± SD, in **B**, **C**, and **D** for group comparison; *n* = 6 biological repeats, except *n* = 4 biological replicates in WT group of 4-HNE. *n* = 6–23; significance calculated by *t* test. *P < 0.05, **P < 0.01, ***P < 0.001.

Based on the FFA lipidomic result from AAV serotype testing described above, we decided to use AAV2 and AAV5 as the candidate vectors for further study. Human *CYP4V2* cDNA driven by a CAG promoter and WPRE enhancer were packaged into AAV2 and AAV5, respectively. Both vectors were transduced into our BCD patient cell-based disease model platforms: BCD iRPE cell lines from 6 individual patients with BCD. A detailed AAV treatment plan is shown in Figure 3A. In brief, we applied a high dose (MOI = 1 × 10^5^ vg/cell) and low dose (MOI = 1 × 10^4^ vg/cell) of each AAV2 and AAV5 vector on mature iRPE cells from patients with BCD that had been cultured for 2 months (60 days). Three months after AAV transfection (150 days in total), we exposed BCD iRPE cell-based disease models from each AAV-treated group to blue light and measured the levels of ROS, 4-HNE, and cell viability to assess the therapeutic effect of each of the 4 AAV treatment strategies. WT iRPE cell lines from 4–6 individual healthy donors were used as a control group.

After blue light exposure, the high dosage of both AAV2 and AAV5 significantly reduced the ROS level in BCD iRPE cells and reduced the ROS levels back to those seen in WT samples. The low dose of AAV2 also significantly reduced ROS levels but not as well as the high-dose treatment. The low-dose AAV5 treatment failed to show any significant decrease in ROS levels in response to blue light treatment (Figure 3B). Next, from the 4-HNE results, all 4 AAV treatment strategies reduced the average 4-HNE concentration in BCD iRPE after blue light exposure, and only low-dose AAV5 treatments failed to show significant reductions. Moreover, none of the 4 AAV treatments reduced the 4-HNE levels to those seen in WT iRPE samples (Figure 3C). The high dose of AAV2 achieved the strongest cell death rescue effect in iRPE samples from 6 patients. Both high-dose AAV2 and AAV5 showed significant rescue effects (Figure 3D).

As negative control to AAV2-*CYP4V2* and AAV5-*CYP4V2* treatments, we applied high doses of AAV2 and AAV5 vectors without *CYP4V2* cDNA packaged (labeled as AAV-null in Supplemental Figure 4) to BCD iRPE cells. After exposure to blue light, there were no significant differences in ROS levels or cell death rates in these cells compared with the ones without any AAV treatment (Supplemental Figure 4).

When we process the data from each AAV treatment group, we noticed the outcome variations among all 6 BCD iRPE cell lines. Then we present the ROS (Figure 3B), 4-HNE (Figure 3C), and cell death rate (Figure 3D) of iRPE in individual patients with BCD from each AAV treatment strategy. The exact value of all the 3 outcome measurements are summarized in Supplemental Table 2. In brief, in BCD-P1, both high and low doses of AAV2 showed similar levels of average ROS reduction. In AAV5, only the high dose of AAV5 significantly reduced ROS levels in BCD-P1. In addition, the high dose of AAV5 reduced ROS levels more than that of AAV2 in BCD-P1. In BCD-P2 and BCD-P3, all 4 treatment strategies significantly reduced ROS levels. In BCD-P4 iRPE cells, all 4 tested AAV treatments reduced average ROS levels slightly; only the high dose of AAV2 showed significant efficacy. All AAV treatments on BCD-P5 iRPE cells significantly reduced average ROS levels. The high-dose AAV2 and AAV5 treatments significantly reduced more ROS levels compared with the low-dosage treatment. In BCD-P6 iRPE cells, the high doses of AAV2 and AAV5 both significantly reduced average ROS levels. Low-dose AAV2 also showed a significant reduction in average ROS levels, but high doses of AAV2 and AAV5 had more significant reductions in average ROS levels compared with the low doses of each respective AAV.

Reduction of 4-HNE concentrations from each AAV treatment had similar trends to those observed in the tests of ROS. In BCD-P1 iRPE cells, AAV5 had the best performance in 4-HNE reduction. In BCD-P2 and BCD-P3, the treatment effective in reducing 4-HNE average level from different treatment are similar. In iRPE cells of BCD-P4, BCD-P5, and BCD-P6, all 4 tested AAV treatments showed significant efficacy, and high doses of AAV2 and AAV5 reduced 4-HNE average levels more than the low doses of each respective AAV.

The high doses of AAV2 and AAV5 each generated a significant reduction in cell death rate in iRPE cells in all 6 patients with BCD except in BCD-P2, where only the high dose of AAV5 generated a significant reduction. BCD-P1 and BCD-P5 iRPE cells showed a more statistically significant reduction in death rates in response to the high-dose AAV5 treatment, while the high dose of AAV2 produced more statistically significant reductions in cell death rates in BCD-P4 and BCD-P6 iRPE cells. In BCD-P2 and BCD-P3, the high doses of AAV5 and AAV2, respectively, provided the strongest efficacy as shown by lower levels in the cell death rates. Notably, in both BCD-P2 and BCD-P3, the best gene therapy treatment strategy reduced the cell death rates to below 10%. Among all the other 4 BCD iRPE cells (BCD-P1, BCD-P4, BCD-P5, and BCD-P6), a reduction in cell death rates to levels below 10% was only observed in BCD-P1’s iRPE in response to both high dose AAV2 and high dose AAV5 treatments. AAV caused reduction of ROS, 4-HNE level, and cell death rate normalized against the value of blue light–induced ROS, 4-HNE level, and cell death rate, respectively, presented in Supplemental Figure 5.

In addition, the average cell death rate after 20–23 replicates were consistent with each other. The metabolite proportions for BCD iRPE cell lines ranged from 10% to 50%. This range may reflect regional differences in human RPE cells cultured in vitro (24).

### Distinct AAV transduction rate from iRPE in individual patients with BCD.

**Figure 4:**
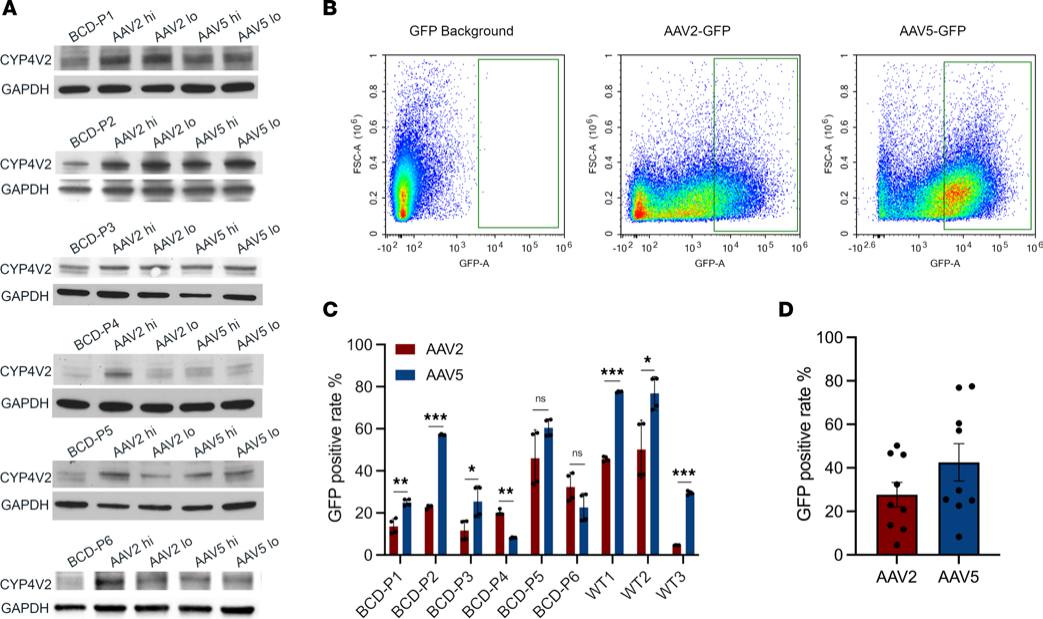
Distinct AAV transduction rate from iRPE of individual patients with BCD. (**A**) Immunoblot results of CYP4V2 expression levels in individual BCD patient iRPE lines in response to each AAV treatment strategy. (**B**) Gating criteria for quantification of GFP-positive iRPE cells. (**C**) Comparison of transduction rate between AAV2 and AAV5 from individual patient iRPE. All data are presented as mean ± SD. *n* = 4 technical repeats, significance was calculated by *t* test. *P < 0.05, **P < 0.01, ***P < 0.001. (**D**) Comparison of transduction rate of AAV2 and AAV5. All data are presented as mean ± SD. *n* = 9 biological repeats. Significance was calculated by *t* test. *P < 0.05, **P < 0.01, ***P < 0.001.

We used 2 methods to determine the AAV transduction rate in BCD iRPE cells. Firstly, we use immunoblots to investigate the expression level of human CYP4V2 protein in BCD iRPE cells from each AAV treatment strategy (Figure 4A). In BCD-P1, BCD-P2, and BCD-P3 iRPE cells, all 4 treatment strategies (both high and low doses of AAV2 and AAV5) produced higher levels of CYP4V2 protein. In BCD P4 iRPE cells, the high dose of AAV2 resulted in an increase of CYP4V2 expression, while the low dose of AAV2 and high dose of AAV5 also increased CYP4V2 expression but at much lower levels than that seen from the AAV2 high dose. In BCD-P5 iRPE cells, AAV2 high dose and AAV5 (both high and low doses) increased CYP4V2 expression. The low dose of AAV2 also resulted in higher CYP4V2 expression but not as obviously as other treatments. In BCD-P6 iRPE cells, the AAV2 high dose showed the most obviously increase in CYP4V2 expression. The AAV2 low dose and AAV5 high and low doses also increased CYP4V2 protein expression.

Secondly, we transfected BCD iRPE cells with AAV2-eGFP and AAV5-eGFP at a MOI = 1 × 10^4^ (vg/cell) and then quantified GFP^+^ iRPE cells by flow cytometry. For each BCD iRPE cell line, fluorescence intensity of GFP more than 1 × 10^3^ (beyond the iRPE GFP background) was counted as a positive cell (Figure 4B). We have tested 9 iRPE samples; 6 of them were BCD iRPE and 3 were WT iRPE cells. Among all tested samples, BCD-P1, BCD-P2, BCD-P3, and all 3 WT iRPE cells’ transduction rates of AAV5 were significantly higher than AAV2 (Figure 4C). In BCD-P4, the AAV transduction rate of AAV2 was higher than that of AAV5, and there was no significant difference in the AAV transduction rates between AAV2 and AAV5 in BCD-P5 and BCD-P6 (Figure 4C). The exact transduction rates of AAV2 and AAV5 for each sample are summarized in Supplemental Table 3. Overall, the average rate of GFP^+^ iRPE cells of the 9 samples tested, in the levels were higher in AAV5 compared with AAV2, however, there was no significant difference in expression between the 2 vectors (Figure 4D).

To further investigate the AAV vector preference, we also transfected AAV2-eGFP and AAV5-eGFP on the same patient (BCD-P1) at 2 different time points. Results demonstrate that the culturing time only affected the amplitude of transduction rate but not the vector serotype preference (Supplemental Figure 6).

### BCD iRPE cells harboring homozygous deletion mutations in the CYP4V2 gene exhibit a more pronounced improvement following AAV-mediated gene augmentation therapy than BCD iRPE cells with homozygous missense mutations in CYP4V2.

**Figure 5:**
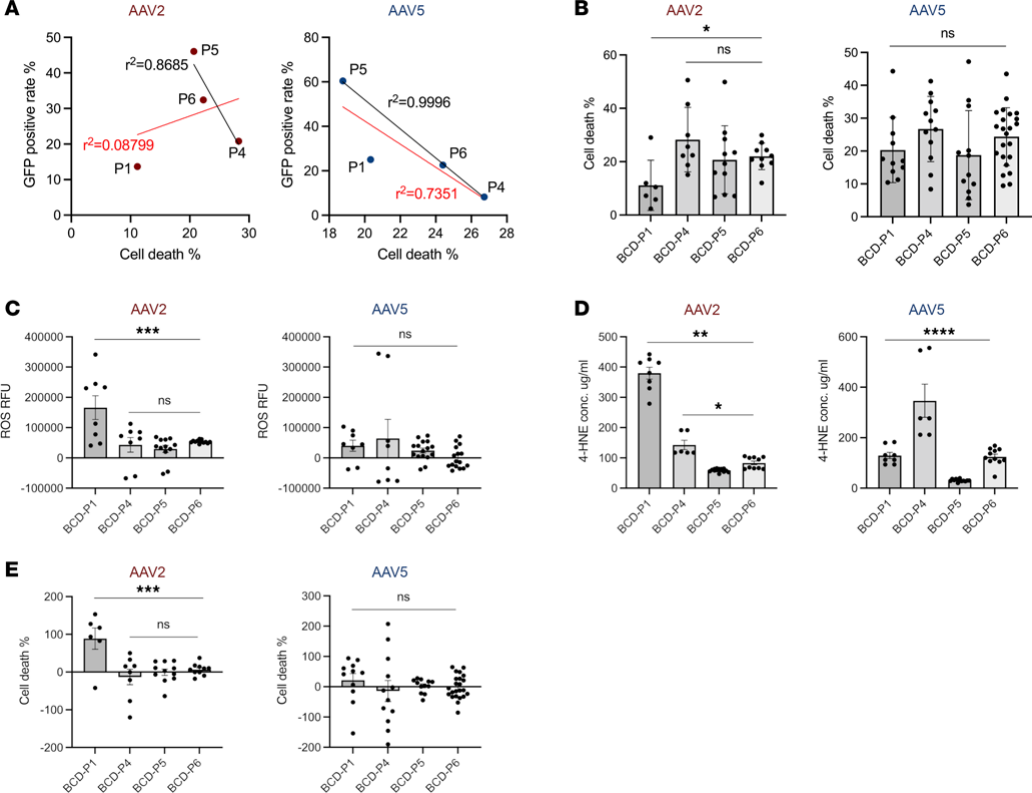
Higher AAV efficacy in homozygous iRPE cells carrying deletion mutation than iRPE of homozygous missense mutations. (**A**) Correlation of AAV transduction rate and cell death rate of iRPE cells with homozygous mutation in *CYP4V2* gene. P1, P4, P5, and P6 are BCD iRPE of homozygous mutations. P1, carries homozygous 17 bp deletion mutation, while P4, P5, and P6 each carry a distinct homozygous missense mutation. For each chart, red linear regression lines contain 4 samples of BCD-P1, -P4, -P5, and -P6, while black linear regression lines contain 3 samples of BCD-P4, -P5, and -P6. *r*^2^ values are shown within each chart. (**B**) Oxidative-induced cell death rate after AAV2 and AAV5 treatment among iRPE cells from patients with homozygous mutation BCD. (**C**–**E**) Changes of ROS (**C**), 4-HNE level (**D**), and cell death rate measurements (**E**) due to AAV2 and AAV5 treatment, normalized by transduction rate, among iRPE cells from patients with BCD with homozygous mutations. In **B**–**E**, data are presented as mean ± SD, *n* = 6–23. Significance was calculated by 1-way ANOVA with multiple comparisons. **P < 0.005, ***P < 0.0005, ****P < 0.0001.

Among the iRPE in 4 homozygous patients with BCD (BCD-P1, BCD-P4, BCD-P5, and BCD-P6) enrolled in this study, BCD-P1 carried a 17 bp deletion mutation, while BCD-P4, BCD-P5, and BCD-P6 carried 3 distinct homozygous missense mutations, respectively (Table 1). From the AAV transduction rate result, BCD-P1 had the lowest AAV transduction rate among all the iRPE in 4 homozygous patients with BCD; however, when we measured the outcomes from each AAV treatment, BCD-P1 had a similar rescue level with others. A regression was done to establish the correlation of AAV transduction rate and iRPE cell death rate with AAV treatments among the iRPE in 4 homozygous patients with BCD (Figure 5A). In AAV2, AAV transduction rates and iRPE cell death rates did not correlate when linear regression analysis was done on all 4 homozygous patients (*r^2^* = 0.08799), but they highly correlated among the 3 homozygous missense patients (*r^2^* = 0.8685). In AAV5, the correlation level of AAV transduction rate and iRPE cell death rate was higher among the 3 patients with BCD with homozygous missense mutations (*r^2^* = 0.9996) compared with the 4 homozygous patients (*r^2^* = 0.7351). Next, we investigated if there was any difference on AAV rescue efficacy among the iRPE in 4 homozygous patients with BCD. We found the cell death rate of BCD-P1 was significantly lower than the iRPE in the other 3 homozygous patients with BCD under the AAV2 treatment, and there was no significant difference among the 3 patients with iRPE with homoxygous missense mutations. In AAV5 treatment, we obtained a similar result; however, there was no significant difference among the 4 homozygous patients (Figure 5B). To validate the difference of AAV rescue efficacies between BCD genotypes with deletion mutation and missense mutations, we calculated the changes of ROS, 4-HNE level, and cell death rates before and after AAV2 and AAV5 treatments on each BCD iRPE sample with homozygous mutations (P1, P4, P5, P6) and then normalized the changes with each their own AAV transduction rates. In AAV2 treatment, BCD-P1 was different from the other 3 patients with missense mutations on all 3 outcome measurements. Due to individual variation in transduction rates of AAV5, there was no statistically significant difference in AAV5 treatments among the 4 patients (Figure 5, C–E).

## Discussion

To successfully translate an in vitro study into a clinical trial, 2 key points should be kept in mind when conducting the bench-to-bedside research: first, the patient cell-based disease model should recapitulate the disease features from patients, and second, outcomes that can be measured in patient cell-based disease models need to be identified. Clinically, BCD is associated with RPE atrophy (7). The eye is the light-sensing organ, and a key function of the RPE is light absorption (25). Light is also a source of oxidative stress to the retina, which should be a key factor to be considered when modeling a retina disease in vitro. However, previously published research on BCD iRPE (10–12, 15) has not considered the effect of photodamage on BCD iRPE. Furthermore, prior studies have not investigated whether BCD gene therapy or other drug candidates can mitigate light-induced RPE cell death (10–12, 15).

Blue light, pervasive in our environment from both sunlight and artificial sources such as office lighting and electronic devices (TVs, computer monitors, smartphones, notebooks, and tablets) poses a significant risk. Our study reveals that blue light exposure significantly induced lethal oxidative stress and increased the cell death rate in iRPE cells from patients with BCD compared with iRPE cells from WT individuals. These findings underscore the critical role of light-induced retinal damage in the development of BCD. Furthermore, this in vitro phenotype in iRPE cells of BCD patients can be accurately measured through cell viability assays, establishing the level of cell death as a strong biomarker and a clinically relevant indicator for evaluating the efficacy of potential treatments, including gene therapy. This insight should guide ophthalmologists to carefully consider the implications of prescribing intensive light exposure tests, such as retinal autofluorescence, for patients with BCD. Additionally, our research has shown that gene therapy, specifically AAV-mediated therapy, can significantly mitigate light-induced cell death in BCD iRPE cells. This constitutes the first proof-of-concept study indicating that AAV-CYP4V2 gene therapy could be an effective treatment for light-induced RPE damage in BCD.

To understand the connection between biochemical abnormalities (such as unusually high levels of PUFAs) and the clinical manifestations (notably, increased cell death) in iRPE cells of patients with BCD, we focused on AA (C20:4, omega-6), the predominant PUFA accumulated in BCD iRPE cells (Supplemental Figure 2A). Our objective was to identify a biomarker directly linked to iRPE cell death. We hypothesized that exposure to blue light could cause the energy from short-wavelength light to interact with the numerous double bonds in the accumulated PUFAs within BCD iRPE cells, leading to lipid peroxidation. The resulting peroxidation products of these PUFAs might be the primary cause of cell death in BCD RPE cells. We considered 2 highly reactive aldehydes produced by PUFA degradation as potential culprits: 4-hydroxy-2-hexenal (4-HHE) (from omega-3 PUFAs) and 4-HNE (from omega-6 PUFAs). Given the significant accumulation of AA (an omega-6 PUFA) in BCD iRPE cells, we measured the levels of 4-HNE after blue light exposure and found abnormally high concentrations correlating with cell death in BCD RPE cells. Consequently, 4-HNE levels can serve as a measurable molecular biomarker to assess the effectiveness of various therapeutic approaches in cell-based disease models specific to patients with BCD.

Our study fills the gaps in previous iRPE studies about patients with BCD (10–12, 15) by examining individual differences in phenotype and response to different AAV-mediated gene therapy treatment strategies among iRPE cells from a diverse pool of 6 patients with BCD of 3 ethnic origins harboring distinct *CYP4V2* mutations. iRPE cells from no more than 3 patients with BCD have been studied and analyzed in any BCD research previously. In previous BCD studies (10–12, 15), all the studied patients were from the single ethnic origin of east Asian (either China or Japan).

To date, over 100 *CYP4V2* mutations have been reported (8); *CYP4V2* mutation carrier frequency study revealed that the prevalence of BCD may have been underestimated, and the most common *CYP4V2* mutations are different among various populations (9). Here, our research timely reported results from iRPE cells of 6 individual patients with BCD of different ethnic origins with distinct *CYP4V2* mutations common in each of East Asian, European, or South Asian populations. Among the 6 patients with BCD, distinct mutations caused various expression levels of CYP4V2 protein in BCD iRPE cells (Figure 1B). Moreover, after exposure to blue light, the individual ROS, 4-HNE level, and cell death levels among all the iRPE cells of 6 patients with BCD were variable and significantly different. The high cell death rate observed in the iRPE cell lines BCD-P1, -P4, -P5, and -P6 (Figure 2) are consistent with prior studies that reported P1’s mutation c.802-8_810del17insGC (deletion of Exon 7) as a severe mutation (7) and reported P4’s mutation homozygous p.Arg400Cys and P6’s mutation homozygous p.Met66Arg to be associated with early onset (7, 19). P5’s mutation p.Ile111Thr is considered to be deleterious (8), but patients harboring homozygous p.Ile111Thr have shown wide phenotype variability, which suggests that environmental or epigenetic factors may affect disease progression (26).

Next, we investigated if the personalized AAV-mediated gene augmentation treatment approach is preferable for individual patients with BCD — namely, we tested whether grouped BCD samples favor the same AAV serotype and dosage. We grouped the 6 individual BCD cellular disease models under each AAV treatment strategy to evaluate the overall therapeutic efficacy. The results show that the high dose of AAV2 was most effective at treating this inherited retinal disorder (Figure 3, B–D). In homozygous BCD iRPE cells, all the 4 cellular models (BCD-P1, BCD-P4, BCD-P5, and BCD-P6) in this study only responded significantly to high-dose treatments (Figure 3, C–D). We also observed that BCD-P1 and BCD-P5 responded the best to a high dose of AAV5, while BCD-P4 and BCD-P6 responded the best to the high dose of AAV2 (Figure 3, B–D). In the iRPE cells of BCD-P2 and BCD-P3, the high dose of AAV2 and the high dose of AAV5 had similar rescue effects. However, BCD-P2 responded best to the high dose of AAV5, while BCD-P3 responded best to the high dose of AAV2 (Figure 3, B–D). Notably, iRPE cells of BCD-P2 responded the most favorably to all tested treatment strategies. The average cell death rates of AAV-treated iRPE cells of BCD-P2 from each AAV-treatment plan were similar, independent of dosage. Our findings demonstrate the importance of analyzing individual responses to treatment as they may differ from the aggregate response to treatment. Additionally, there are also personalized response to AAV vector serotypes among BCD patient–specific iRPE cellular models. To further validate the individual preference for various AAV vectors, we assessed the AAV transduction efficiency in the iRPE cells of each patient. Using CYP4V2 immunoblots and AAV-eGFP (for serotypes AAV2 and AAV5), we confirmed the personalized responses of BCD patient–specific iRPE cells to different AAV vectors (Figure 4). These findings indicate that patient-specific stem cell–derived cellular models, such as iRPE cells, hold the potential to be utilized to screen the most effective gene therapy vector for an individual patient. This approach supports the development of precision gene therapy, taking advantage of the response of each patient for specific AAV vectors at the cellular level.

Among all 4 patients with BCD with a homozygous mutation in their *CYP4V2* gene, BCD-P1 carries the most common mutation in the *CYP4V2* gene: a 17-base deletion in exon 7 (6, 9), while BCD-P4, -P5, and -P6 carry 3 distinct missense mutations, respectively. Oxidative stress–induced ROS, 4-HNE concentration and cell death rates in all 4 homozygous BCD patient iRPE cells were similar (Figure 2). Interestingly, compared with the other 3 homozygous models (BCD-P4, BCD-P5, and BCD-P6), the oxidative stress–induced phenotype in BCD-P1’s iRPE was more readily reversed by AAV-mediated gene augmentation therapy (Figure 5). Even though the AAV transduction rate of BCD-P1 was lower than the other 3 homozygous BCD iRPE cells (Figure 4C), cell death in BCD-P1 iRPE cells was rescued the most by AAV treatment compared with the other homozygous BCD iRPE cell lines. In the AAV2 treatment, BCD-P1 had a greater reduction in all the 3 outcome biomarkers compared with the iRPE in the other 3 homozygous patients with BCD (BCD-P4, -P5, -P6) (Figure 5). As autosomal recessive genetic disorders, usually the null phenotype is expected to be found in cellular or molecular level. However, scientists have found, in CNGB3-associated recessive achromatopsia, certain disease-associated mutations caused “gain-of-function” alterations (27). In our study, homozygous missense mutations in BCD-P4, BCD-P5, and BCD-P6 led to “gain-of-function” phenotypes by interference with the multimerization of transgenic *CYP4V2*. In contrast, the homozygous deletion mutation in BCD-P1 caused a “loss-of-function” null phenotype that may be more easily corrected by gene augmentation therapies.

Currently, gene therapy is increasingly acknowledged by the Centers for Medicare & Medicaid Services (CMS) as a promising method for treating monogenic disorders. One of the most significant challenges that scientists and physicians face is the variability in responses from individual patients, often referred to as chemical individuality (28–31). Despite this, the prevalent strategy for developing gene augmentation therapies still relies on a “1 vector” and “1 dose” approach for all patients diagnosed with the same condition. Our study reveals that the diseased tissues of individual patients responded differently to AAV vectors. This diversity leads to varied patient responses to specific gene therapy treatments, including the use of different vectors and doses. However, our study faces limitations, primarily due to the limited number of BCD patient cell lines analyzed (6 individuals). Ideally, further research should expand the collection and analysis of iRPE cells from a broader cohort of patients with BCD, even though amassing a large sample size is a well-known challenge in the study of rare diseases. Furthermore, to investigate if the phenotype differences and distinct response among patients to a specific AAV treatment strategy is due to mutation differences or other individual variability among patients, future studies should enroll not only patients of divergent mutations but also multiple patients for each specific mutation; this may be feasible for more common disorders. Finally, considering the systemic immune response and other in vivo features relating the communication among different tissues, the in vitro testing on single type of cells cannot completely reflect the actual in vivo environment. The differences we observed in our in vitro transduction studies suggest the need to better understand the variability of individuals within the monogenic inherited retinal disorders.

Taken together, adopting a precision medicine approach to determine the most effective therapeutic vector and dosage for each individual could be crucial for enhancing treatment outcomes, even before an IND application for phase 1 trials. Our findings support the method of testing candidate vectors on cellular models derived from the patient’s stem cells before potential translation of the treatment strategy to patients. Hence, clinical trials could be improved by recruiting patients whose cells have undergone testing in culture and have demonstrated favorable responses to specific candidate vectors. Optimization of vector and dosage in patient cells should improve the efficacy of gene therapies for genetic disorders, validating the principle and core value of precision medicine: designing the right dose of the right vector for the right person.

## Methods

### Sex as a biological variable.

Our study contained samples from both men and women. In all reported data, sex was not considered as a biological variable, and findings for both sexes were similar.

### Generation of iRPE cells from patients with BCD and WT individuals.

Skin fibroblast cells from 6 patients with BCD, and their age- and sex-matched WT control donors were each plated and cultured in a 12-well plate until the cells became adherent and approximately 70%–80% confluent. The culture medium was then removed and the cells were transfected with CytoTune-iPS 2.0 Sendai Reprogramming Kit (A16517, Invitrogen) as previously described (32–34).

iPSC differentiation started at passage 3 to 6 for all iPSC lines of patients with BCD and healthy controls according to previously published protocols (35). In brief, iPSC colonies were cultured to confluence in 6-well culture dishes (Costar, CORNING) pretreated with 1:50 diluted Matrigel (CORNING, 356230) in differentiation medium consisting of KO DMEM (Thermo Fisher Scientific, 10829018), 15% KO serum replacement (Thermo Fisher Scientific, 10829028), 1% nonessential amino acids (Thermo Fisher Scientific, 11140050), 2 mmol/L glutamine (Thermo Fisher Scientific, 35050061), 50 U/mL penicillin-streptomycin (Thermo Fisher Scientific, 10378016), and 10 mmol/L nicotinamide (Sigma-Aldrich, N0636) for the first 14 days. During days 15–28 of differentiation, medium was supplemented with 100 ng/mL human Activin-A (PeproTech, 120-14). From day 29, Activin-A was removed until differentiation was completed. After 8–10 weeks, pigmented clusters formed and were manually picked and plated on Matrigel-coated dishes. Those cells were maintained in RPE culture medium; MEMα modification–based (Sigma-Aldrich, M-4526) medium, which contains N1 supplement (5 mL per 500 mL medium); taurine (125 mg per 500 mL medium); hydrocortisone (10 μg per 500 mL medium); and triiodo-thyronin (0.0065 μg per 500 mL medium) (all from Sigma-Aldrich) as well as 2 mmol/L glutamine, 50 U/mL penicillin-streptomycin, 1% nonessential amino acids, and 5% FBS (all from Thermo Fisher Scientific) and cultured for another 6–8 weeks to allow them to form a functional monolayer or longer periods as specified below before testing for functional assays (35, 36).

The RPE cells differentiated from the iPSCs of patients with BCD were observed under light microscopy, and distinct RPE pigment and hexagonal cell shapes were seen (Supplemental Figure 1). In addition to morphological distinctions, iRPE cells from patients with BCD were also validated by the presence of mature RPE-specific markers RPE65 and CRALBP.

### Construction and production of recombinant AAV vectors.

AAV vectors encoding human CYP4V2 protein (AAV-CYP4V2) vectors tested in this study were provided by Reflection Biotechnologies and were custom made by Vector Biolabs and the Viral Vector Core of Andelyn Biosciences. A human CYP4V2 cDNA or a codon-optimized cDNA (ordered from GenScript by Reflection Biotechnologies) encoding human CYP4V2 protein (NP_997235.3) was packaged in the AAV-CYP4V2 vectors. See Supplemental Table 1 for detailed information of AAV vectors used in this study. The AAV2-CYP4V2 and AAV5-CYP4V2 vectors used in generating the immunoblots, ROS, 4-HNE level, and cell viability results shown in this paper were manufactured by Andelyn Biosciences. AAV-CYP4V2 vector productions were accomplished using a 3-plasmid transfection method in HEK 293 cells (37). AAV2-null and AAV5-null vectors (provided by Reflection Biotechnologies and produced by Vector Biolabs) that do not express any transgene were used as negative control. AAV2-eGFP and AAV5-eGFP were purchased from Andelyn Biosciences by Reflection Biotechnologies.

### Transduction of AAV vectors in BCD iRPE cells.

iRPE cells derived from patients with BCD were transfected with various AAV vectors described herein in serum-free RPE medium. After 1 day, the virus-containing medium was replaced with fresh serum–containing RPE medium to continue RPE culture. To assess the therapeutic effects of different dosages, different multiplicities of infection (MOI) were tested. For iRPE samples used in the untargeted lipidomic assay of FFAs, the AAV treatment was applied during the sixth week of culturing; then, FFA were tested during the 4 weeks after AAV transfection (week 10 of culturing). For iRPE samples used in outcome measurement of ROS, 4-HNE level, and cell viability, AAV treatments were applied according to the treatment plan presented in Figure 3A.

### Establishment of BCD isogenic control cell lines using the CRISPR/Cas9 system.

The BCD P1 isogenic control cell line, which repaired the c.802-8_810del17insGC mutation in the *CYP4V2* gene in BCD P1 iPSC, was generated by CRISPR/Cas9 gene editing approach. DeskGen software was used to design the guide RNA. The guide RNA (Custom Alt-R CRISPR-Cas9 sgRNA, Integrated DNA Technologies) and Cas9 protein (1081060, Integrated DNA Technologies) in a ribonucleoprotein (RNP or protein-RNA) complex and the donor template (Integrated DNA Technologies) were used to correct/repair the c.802-8_810del17insGC mutation in the *CYP4V2* gene in BCD-P1 iPSC to create the isogenic control of P1. Two million iPSCs from BCD-P1 were electroporated with 30 pmol Cas9 protein + 270 pmol single-guide RNA + 10–20 μg single-stranded DNA oligonucleotides donor for a 100 μL final reaction volume (in reaction buffer from Lonza kit P3) in Lonza 4D Nucleofector (Lonza, V4XP-3032). Afterward, iPSCs that survived from nucleofection were sorted in single-cell expansion and were then sequenced to identify the homologous genetically repaired iPSCs no longer harboring the c.802-8_810del17insGC mutation, which were then differentiated into iRPE cells. Sequence information of gRNA, donor template, and primer sequences used for amplifying the CYP4V2 homology-directed repair (HDR) site were summarized in Supplemental Table 4.

### Immunoblots.

Cells were lysed in M-PER mammalian protein extraction reagent buffer (Pierce, 78501) containing proteinase inhibitors (Roche, 11836170001). Total protein was quantified using a Bio-Rad protein reader. Protein samples (35 μg/sample per lane) were then separated on a 10% Tris-Cl gradient gel and electroblotted onto a nitrocellulose membrane. Membranes were incubated in blocking buffer for 1 hour at room temperature, washed 3 times in PBS + 0.1% Tween-20 for 10 minutes each, and then incubated with the primary antibody in blocking buffer overnight at 4°C. Primary antibodies against the following proteins were used for Western blots: CYP4V2 (rabbit polyclonal, 1:1500, MilliporeSigma, SAB1410565); RPE65 (mouse monoclonal, 1:7,500, Novus Biologicals, NB100-355); CRALBP (rabbit polyclonal, 1:10,000, Abcam, ab15051); and GAPDH (mouse monoclonal, 1:5,000, Abcam, ab9485). Anti-mouse and anti-rabbit secondary antibodies were obtained from Abcam (ab99697 and ab6728) and used at a concentration of 1:5,000 for CYP4V2 and 1:20,000 for the others.

### Untargeted FFA liquid chromatography mass spectrometry (LC-MS).

This assay was performed at the Columbia University Medical Center (CUMC) biomarker core facility. In brief, FFAs were chloroform-methanol extracted. After culturing for 10 weeks (both AAV-treated iRPE cells and nontreated iRPE cells), about 1 million iRPE cells were homogenized in 150 μL water, and 100 μL of homogenate was mixed with 3 mL chloroform/methanol (v/v = 2:1) containing internal standards (Palmitic acid-D31, C12 ceramide, C25 ceramide, C17 sphingosine, C17 sphinganine). The sample was vortexed well, and 0.5 mL of water was added to allow for phase separation. The mixture was vortexed again and centrifuged at 3,000*g* for 10 minutes at 4°C. The lower organic phase was transferred to a clean glass tube using a Pasteur pipette. In total, 2 mL of chloroform was added to the residual aqueous phase, followed by vortex mixing and centrifugation (3,000*g*) to extract any remaining lipids. The lower organic phases were pooled and evaporated under nitrogen at 37°C. The extracted lipids were reconstituted in 50 μL methanol/acetonitrile (v/v = 1:1) and transferred to LC autosampler vials for injection. All assays were performed on a Waters Xevo TQ MS ACQUITY UPLC system (Waters Corporation). FFAs were eluted using a 100 mm Waters ACQUITY UPLC HSS C18 column and monitored using the negatively selected ion recording (SIR) method.

### Blue light exposure.

iRPE cells were seeded in 96-well black/clear-bottom plate. After designed time points, they were exposed to 430 ± 20 nm (blue) light at 1.5 mW/cm^2^ for 25 minutes for iRPE cultured in 96-well black/clear bottom plate in PBS (+) containing 10 μg/mL glucose. The same seeding density was used for all cell lines. After blue light exposure, treated cells were fed with fresh RPE medium and recovered in the incubator of 5% CO_2_ and 37°C overnight; then, all the cells were processed for outcome measurements.

### ROS assay.

To observe the effect of blue light exposure on ROS/superoxide levels (a measurement of oxidative stress) on iRPE cells, we probed ROS on iRPE samples of WT and patients with BCD (both untreated and AAV treated) using the ROS Detection Cell-Based Assay Kit (DHE) (Cayman Chemical, 601290). In brief, RPE cells cultured in black/clear bottom 96-well plate (Thermo Fisher Scientific, 165305) were loaded with 10 μM Superoxide Detection Reagent, a cell-permeable probe that reacts with superoxide to produce a red fluorescent product. Next, the RPE samples were divided into 2 groups. Group 1 was exposed to blue light for 25 minutes while group 2 was kept at room temperature without exposure to blue light. Then all samples were washed by ROS washing buffer carefully 3 times. The plate was read using a fluorescence reader (Molecular Devices, SpectraMax iD3) at an excitation wavelength of 485 nm and an emission wavelength of 600 nm.

### Lipid peroxidation (4-HNE) assay.

Total protein from 5 × 10^6^ iRPE cells per testing sample was extracted and normalized as described above. Samples were tested by Lipid Peroxidation (4-HNE) Assay Kit (Abcam, ab238538) for detection and quantification of 4-HNE protein adducts. Assays were performed according to the protocol provided by the kit manufacturer.

### Cell viability assay.

Live/healthy iRPE cells were labeled by cell-permeant dye Calcein AM (Thermo Fisher Scientific, C3099) at a final concentration of 3 μmol/mL PBS(+), and dead/sick cells were labeled by PI (Thermo Fisher Scientific, P3566) at a final concentration of 2 μg/mL PBS(+) at room temperature for 1 hour. Since PI is DNA binding and it is not permeant to live cells, it is commonly used to detect dead cells. Then after washing with PBS(–), cellular fluorescence levels were observed, and photos were taken using an inverted fluorescence microscope (Nikon Eclipse Ts2R) at ×20 magnification. Dead/live cell numbers were calculated after photos were processed by ImageJ (Fiji; NIH). For quantification of the results, all images were processed using ImageJ (representative processing image shown in Supplemental Figure 7) as follows: (a) Open image: image type 8 bit, table: red/green; (b) FFT bandpass filter range 3–40 pixels, tolerance of direction: 5%; (c) threshold adjust by default and watershed applied; and (d) analyze particles by size from 100 (red/dead cells) or 300 (green/live cells) to infinity (pixels^2^)

### Flow cytometry.

iRPE were dissociated with 0.05% trypsin for 20 minutes (Thermo Fisher Scientific, 25300054) and resuspended in HBSS (Thermo Fisher Scientific, 14025092) with 1 μg/mL DAPI. Green fluorescent positive cells were detected by fluorescence channel FITC. DAPI positive cells were detected by fluorescence channel Brilliant Violet 421. Data were acquired on cell sorter Sony MA900 and analyzed with NovoExpress Version 1.5.6.

### Statistics.

Statistical analyses were performed by GraphPad Prism. An unpaired 2-tailed Student’s *t* test was used for comparison between any 2 groups analyzed. One-way ANOVA with Tukey’s test and Welch ANOVA with multiple comparisons were used for the comparison of more than 2 groups. *P* values of less than 0.05 were considered statistically significant. All data are presented as the mean ± SD.

### Study approval.

Six individual patients with BCD and individual WT healthy donors were enrolled under Columbia University IRB protocol AAAF1849. Written informed consent from participants was obtained.

### Data availability.

The exact results of all the 3 outcome measurements — ROS, 4-HNE level, and cell death rate — from all 6 BCD individual subjects’ iRPE under different treatment groups (including no treatment) are shown in Supplemental Table 2. Exact transduction rate percentage of AAV2 and AAV5 on each sample (6 individual BCD iRPE samples and 3 individual WT control iRPE samples) is summarized in Supplemental Table 3. Values for all data points in graphs are reported in the Supporting Data Values file. All requests for raw and analytical data will be reviewed by the corresponding author and a ReflectionBio delegate to determine if there are any intellectual property or confidentiality restrictions. Any data that can be shared will be released via a data use agreement.

## Author contributions

YL and RRY are co–first authors, listed alphabetically. Conceptualization was contributed by RRY and SHT. Methodology was contributed by YL, RRY, CWH, YSL, and MZCR. Investigation was contributed by YL, CWH, SHT, MZCR, JRS, and LAJ. Data analysis was contributed by YL, YK, SHT, and RRY. Writing of the original draft was contributed by YL, RRY and SHT. Review and editing were contributed by YL, SHT, RRY, LAJ, YSL, CWH, YK, MZCR, and JRS. Resources were contributed by RRY, SHT, and JRS. Supervision was contributed by SHT.

## Supplementary Material

Supplemental data

Unedited blot and gel images

Supporting data values

## Figures and Tables

**Table 1:**
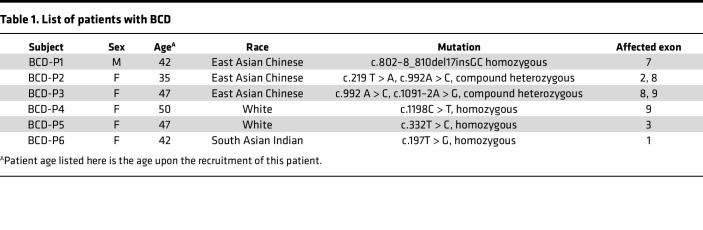
List of patients with BCD

## Version 1 08/22/2024

Electronic publication

## References

[1] Xue K, MacLaren RE (2018). Ocular gene therapy for choroideremia: clinical trials and future perspectives. Expert Rev Ophthalmol.

[2] Cowan CS (2020). Cell types of the human retina and its organoids at single-cell resolution. Cell.

[3] Ji C (2019). Investigation and restoration of BEST1 activity in patient-derived RPEs with dominant mutations. Sci Rep.

[4] Tsai YT (2021). Impaired cholesterol efflux in retinal pigment epithelium of individuals with juvenile macular degeneration. Am J Hum Genet.

[5] Vasireddy V (2013). AAV-mediated gene therapy for choroideremia: preclinical studies in personalized models. PLoS One.

[6] Li A (2004). Bietti crystalline corneoretinal dystrophy is caused by mutations in the novel gene CYP4V2. Am J Hum Genet.

[7] Halford S (2014). Detailed phenotypic and genotypic characterization of bietti crystalline dystrophy. Ophthalmology.

[8] Garcia-Garcia GP (2019). Current perspectives in Bietti crystalline dystrophy. Clin Ophthalmol.

[9] Hanany M (2023). An in-depth single-gene worldwide carrier frequency and genetic prevalence analysis of CYP4V2 as the cause of Bietti crystalline dystrophy. Transl Vis Sci Technol.

[10] Zhang Z (2020). PSCs Reveal PUFA-provoked mitochondrial stress as a central node potentiating RPE degeneration in Bietti’s crystalline dystrophy. Mol Ther.

[11] Hata M (2018). Reduction of lipid accumulation rescues Bietti’s crystalline dystrophy phenotypes. Proc Natl Acad Sci U S A.

[12] Wang JH (2022). AAV2-mediated gene therapy for Bietti crystalline dystrophy provides functional CYP4V2 in multiple relevant cell models. Sci Rep.

[13] Ma Z (2022). A Bietti crystalline dystrophy mouse model shows increased sensitivity to light-induced injury. Int J Mol Sci.

[14] Lockhart CM (2014). Generation and characterization of a murine model of Bietti crystalline dystrophy. Invest Ophthalmol Vis Sci.

[15] Jia R (2023). AAV-mediated gene-replacement therapy restores viability of BCD patient iPSC derived RPE cells and vision of Cyp4v3 knockout mice. Hum Mol Genet.

[16] Yang RR (2023). A patient advocating for transparent science in rare disease research. Orphanet J Rare Dis.

[17] Jiao X (2017). Identification and population history of CYP4V2 mutations in patients with Bietti crystalline corneoretinal dystrophy. Eur J Hum Genet.

[18] Xiao X (2011). Identification of CYP4V2 mutation in 21 families and overview of mutation spectrum in Bietti crystalline corneoretinal dystrophy. Biochem Biophys Res Commun.

[19] Lockhart CM (2018). Longitudinal characterisation of function and structure of Bietti crystalline dystrophy: report on a novel homozygous mutation in *CYP4V2*. Br J Ophthalmol.

[20] Kelly EJ (2011). Finding homes for orphan cytochrome P450s: CYP4V2 and CYP4F22 in disease states. Mol Interv.

[21] Nakano M (2012). CYP4V2 in Bietti’s crystalline dystrophy: ocular localization, metabolism of ω-3-polyunsaturated fatty acids, and functional deficit of the p.H331P variant. Mol Pharmacol.

[22] Tanito M (2009). High levels of retinal membrane docosahexaenoic acid increase susceptibility to stress-induced degeneration. J Lipid Res.

[23] Dalleau S (2013). Cell death and diseases related to oxidative stress: 4-hydroxynonenal (HNE) in the balance. Cell Death Differ.

[24] Ortolan D (2022). Single-cell-resolution map of human retinal pigment epithelium helps discover subpopulations with differential disease sensitivity. Proc Natl Acad Sci U S A.

[25] Strauss O (2005). The retinal pigment epithelium in visual function. Physiol Rev.

[26] Garcia-Garcia GP (2018). Identification of novel CYP4V2 genotypes associated with Bietti crystalline dystrophy and atypical anterior segment phenotypes in Spanish patients. Acta Ophthalmol.

[27] Bright SR (2005). Disease-associated mutations in CNGB3 produce gain of function alterations in cone cyclic nucleotide-gated channels. Mol Vis.

[28] Childs B (1970). Sir Archibald Garrod’s conception of chemical individuality: a modern appreciation. N Engl J Med.

[29] Garrod AE (1975). The Lancet. The incidence of alkaptonuria, a study in chemical individuality. Nutr Rev.

[30] Rosenberg LE (2008). Legacies of Garrod’s brilliance. One hundred years--and counting. J Inherit Metab Dis.

[31] Scriver CR (2001). Garrod’s foresight; our hindsight. J Inherit Metab Dis.

[32] Li Y (2016). Skin biopsy and patient-specific stem cell lines. Methods Mol Biol.

[33] Li Y (2014). Gene therapy in patient-specific stem cell lines and a preclinical model of retinitis pigmentosa with membrane frizzled-related protein defects. Mol Ther.

[34] Li Y (2017). Patient-specific mutations impair BESTROPHIN1’s essential role in mediating Ca(2+)-dependent Cl(-) currents in human RPE. Elife.

[35] Idelson M (2009). Directed differentiation of human embryonic stem cells into functional retinal pigment epithelium cells. Cell Stem Cell.

[36] Sonoda S (2009). A protocol for the culture and differentiation of highly polarized human retinal pigment epithelial cells. Nat Protoc.

[37] Heller KN (2013). AAV-mediated overexpression of human α7 integrin leads to histological and functional improvement in dystrophic mice. Mol Ther.

