# Supplementary material for: Evaluating precision medicine approaches for gene therapy in patient-specific cellular models of Bietti crystalline dystrophy
Source: JCI Insight. 2024 Jul 16;9(16):e177231. doi: 10.1172/jci.insight.177231 (PMC11343589; doi:10.1172/jci.insight.177231)
Supplement: Supplemental data [file jciinsight-9-177231-s241.pdf]

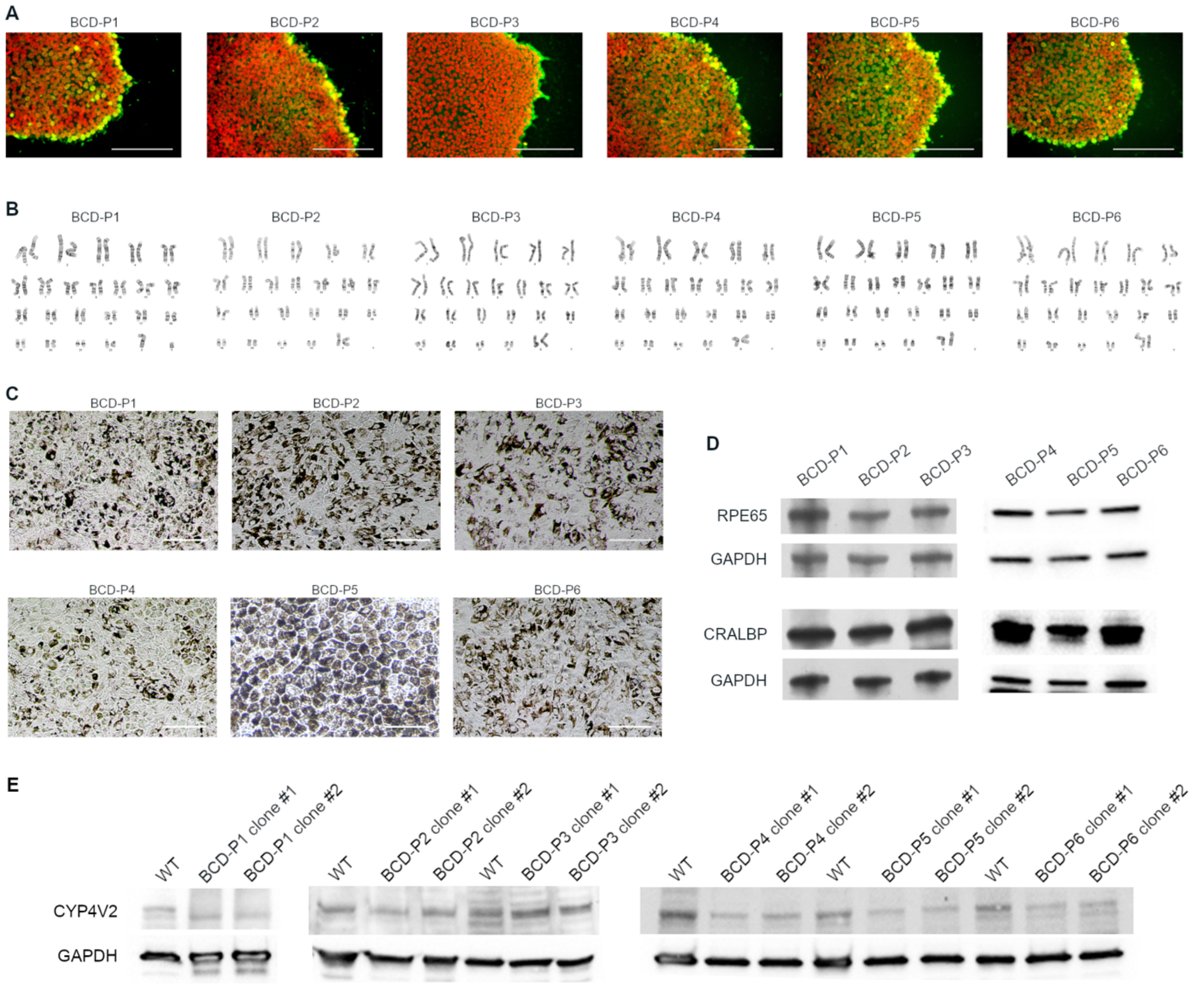

Supplemental Figure1. Cell fate validation and CYP4V2 expression in BCD iRPE. (**A**) Representative immunofluorescence images of pluripotency markers in established BCD patient-specific iPSC lines. Red signals represent NANOG and green signals represent Tra-1-60. Scale bar: 50um. (**B**) Karyotype result of all six BCD patient-specific iPSC lines. All the BCD iPSC lines have normal chromosome karyotype. (**C**) Light microscopy images of BCD iRPE cells. All BCD iRPE present classic human RPE cells morphology, including pigment, hexagonal shape and tight junction between cells. Scale bar: 50um. (**D**) Immunoblot analysis of mature human RPE marker RPE65 (65 kDa) and CRALBP (36 kDa) in iRPE cells from all six BCD patients. GAPDH serves as the loading control. (E) Immunoblot of CYP4V2 expression in multi-clones of each BCD individual iRPE samples. GAPDH serves as a loading control.

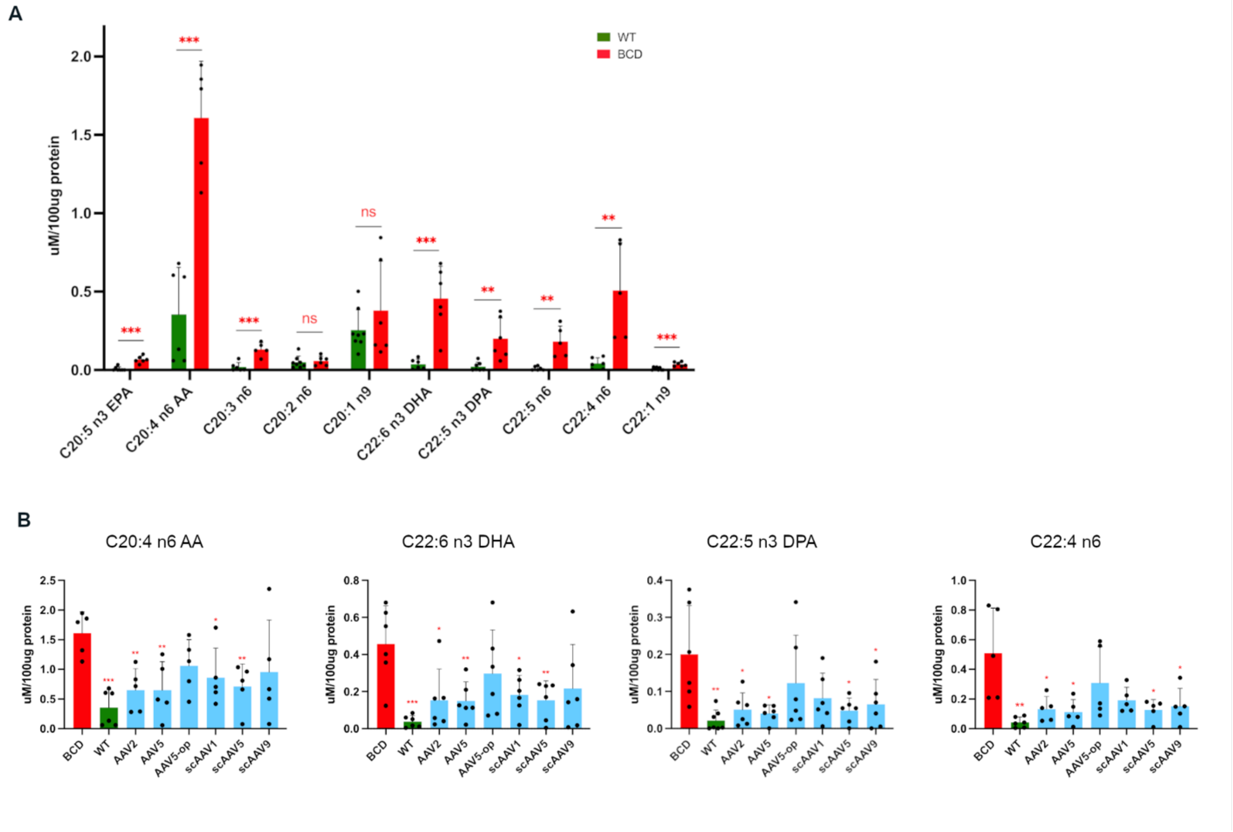

Supplemental Figure 2. AAV-*CYP4V2* reduced major omega-6 and omega-3 PUFAs levels in CYP4V2 patient iRPE cell lines. (**A**) Untargeted lipidomic quantification results show that medium-chain poly-unsaturated fatty acid accumulated in BCD iRPEs. Arachidonic acid (AA) had the highest levels compared to other detected PUFAs. Both omega-3 (n3) and omega-6 (n6) PUFA of 20-Carbon and 22-Carbon are significantly higher in BCD iRPEs compared with WTs. (**B**) Lipidomic quantification shows AAVs can reduce major accumulated PUFA levels in BCD iRPE cells. iRPE treated with AAV2 and AAV5 had the lowest levels of PUFAs among all tested serotypes of AAVs compared to untreated BCD cells. Data presented as mean ± SD, n=5-8, ***P<0.001, **P<0.01, *P<0.05.

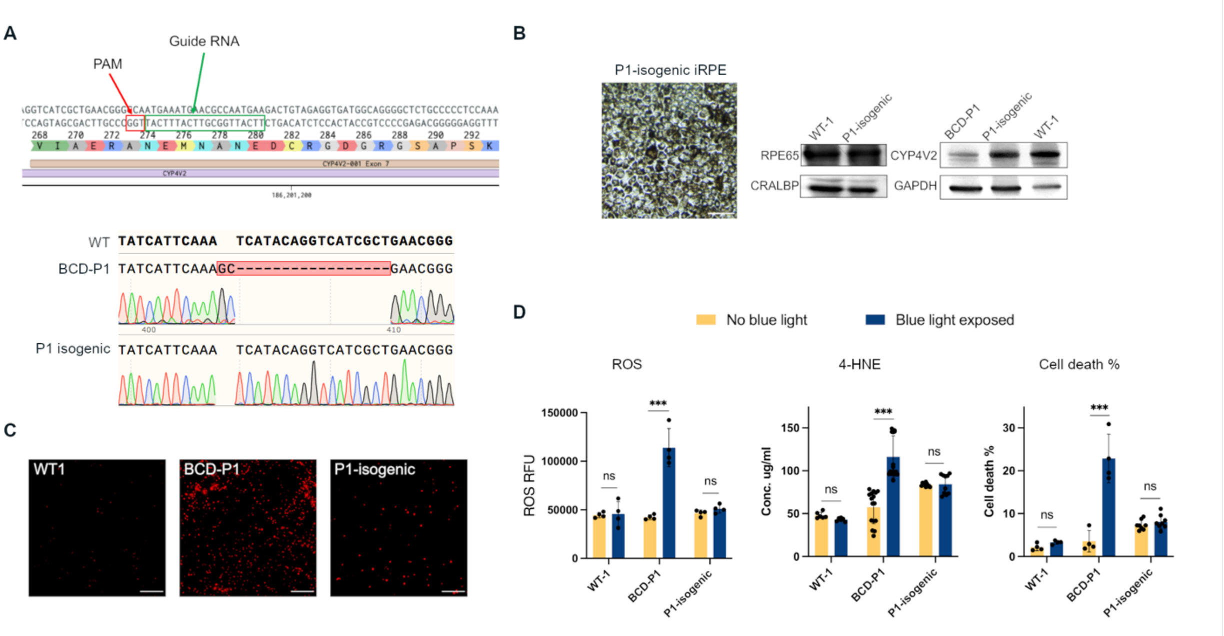

Supplemental Figure 3. CRISPR-mediated genetic repair of the c.802-8_810del17insGC mutation in the *CYP4V2* gene and rescued cellular phenotypes in the genetically repaired P1 isogenic iRPE cells. (**A**) Upper scheme image showed PAM and gRNA binding site of *CYP4V2* gene. The CRISPR guide RNA and Cas9 protein were provided in a ribonucleoprotein (RNP) complex. Lower image of dideoxy sequencing of BCD Patient 1 iPSCs and the corresponding isogenic control iPSC line, indicating a repair of the parental homozygous mutations in isogenic iPSC line. Top panel labeled with WT as CYP4V2 reference sequencing. (**B**) Left: Representative brightfield images showed typical human RPE cellular morphology of isogenic control iRPEs. Scale bar: 20um. Middle: expression of mature RPE markers RPE65 and CRALBP in isogenic control iRPE. Right: CYP4V2 protein expression in iRPEs from WT donor, BCD-P1, and CRISPR repaired BCD-P1 isogenic line. (**C**) Representative fluorescence microscopy images of propidium iodide (red) labeled iRPE cell death status after blue light exposure, cell line labeled within each image, red signal indicated dead cells. Scale bar: 20um. (**D**) Quantification of ROS, 4-HNE level, cells death rate in iRPEs from WT donor, BCD-P1 and BCD-P1 isogenic control line. Data are presented as mean ± SD, n=4-16, significance calculated by t-test, ***P<0.001.

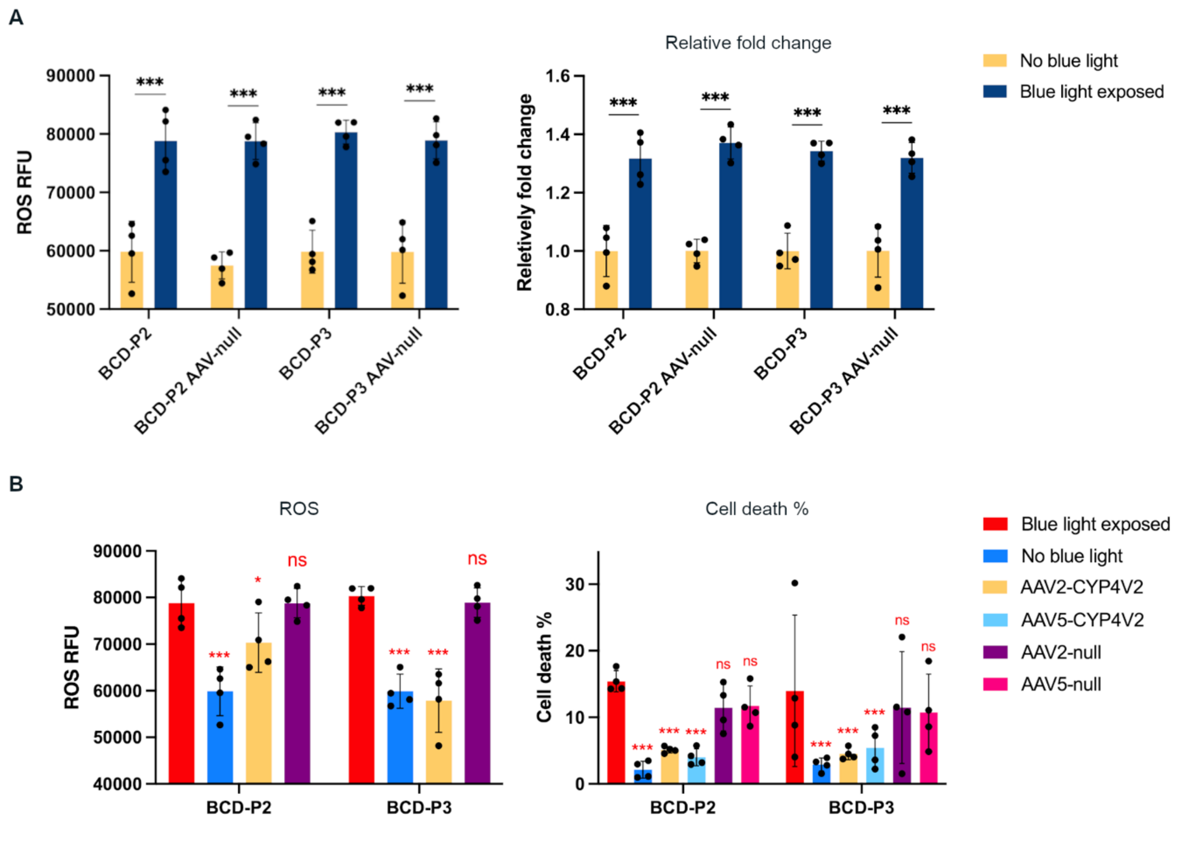

Supplemental Figure 4. AAV2-null and AAV5-null failed to show therapeutics effect. (**A**) Left chart: AAV2-null failed to mitigate the increased ROS level caused by blue light exposure in BCD-iPREs. Right chart: ROS relatively changes before and after blue light exposure in BCD iRPEs and AAV2-null treated BCD iRPEs are similar. (**B**) Left chart: AAV2-null failed to reduce ROS level in BCD iRPEs compared with AAV2-CYP4V2. Right chart: AAV2-null and AAV5-null failed to rescue cell death caused by blue light exposure in BCD-iRPEs. All data presented as mean ± SD, n=4, ***P<0.001, *P<0.05.

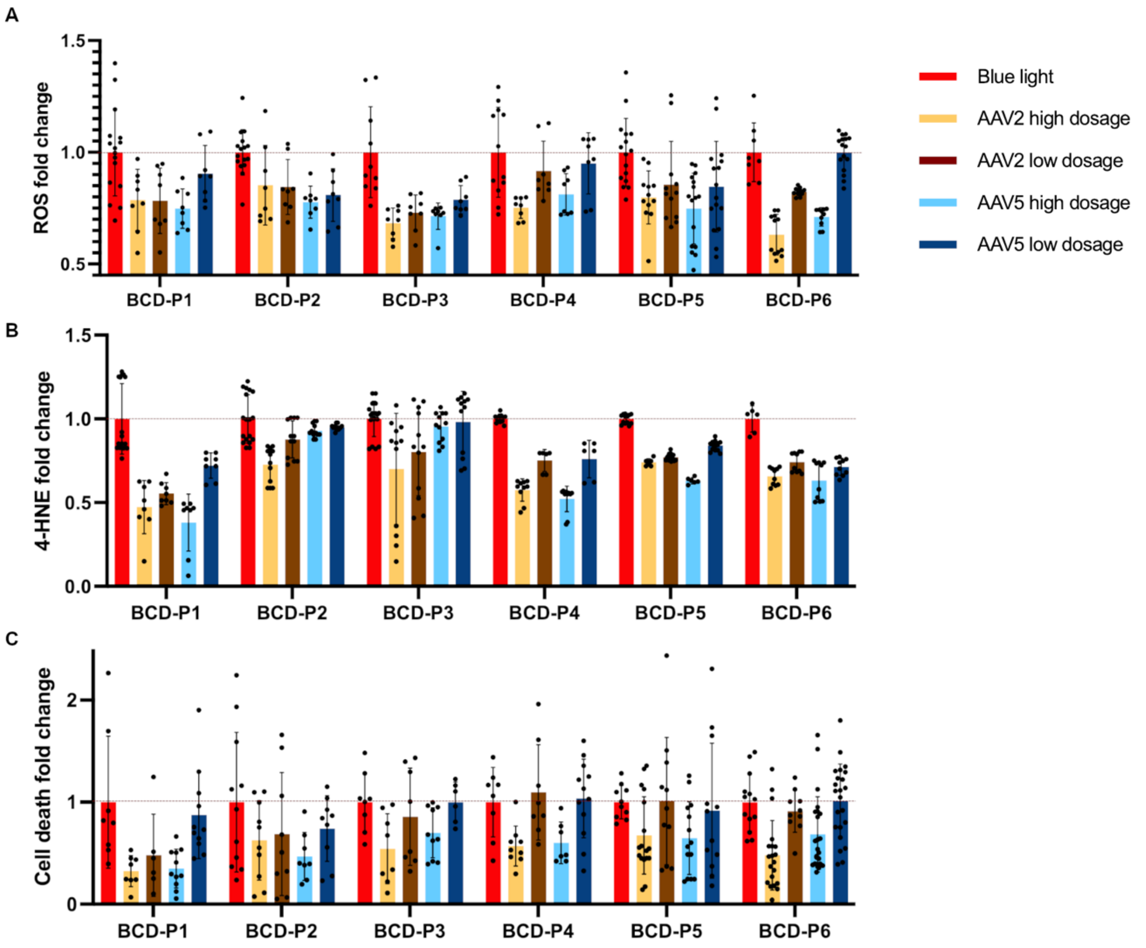

Supplemental Figure 5. Normalized outcome measurements fold changes of ROS, 4-HNE and cell death rate from different AAV treatment. Outcome values of each biomarker, (**A**) ROS, (**B**) 4-HNE, (**C**) cell death rate, are normalized against the values of blue light exposure without any treatment. All data presented as mean ± SD, n=6-23.

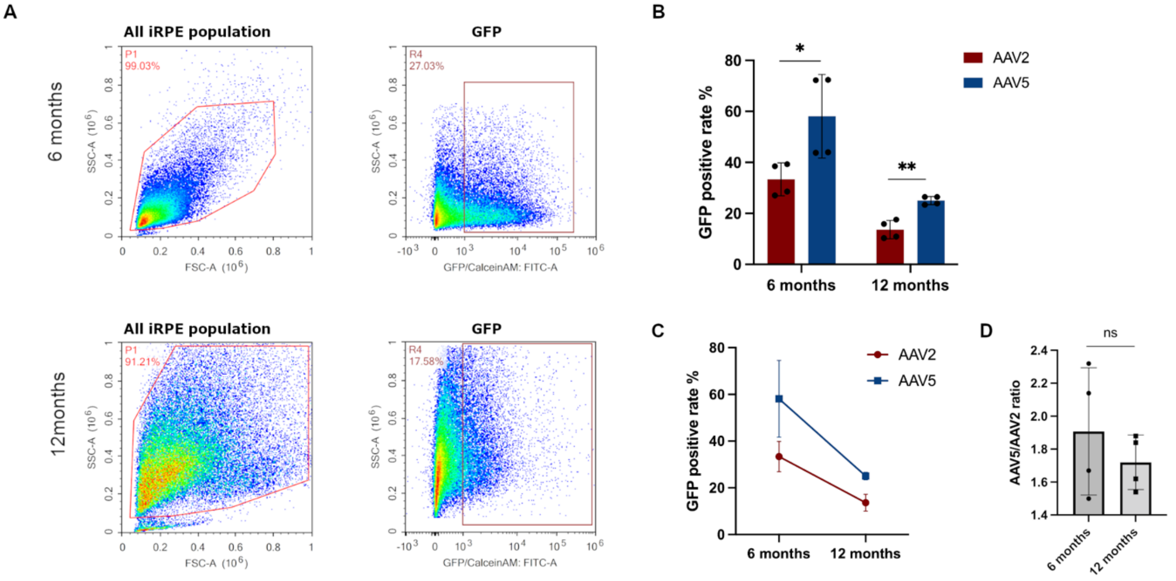

Supplemental Figure 6. In vitro culture time of BCD iRPE affects AAV transduction rate, but does not affect the personalized AAV serotype preference. (**A**) Representative gating images of BCD-P1 at 6-month culturing (upper panel) and 12-month culturing (lower panel). Left panel images represent all the iRPE cells tested by FACS. Compared with 6-month cultured iRPE, longer culturing time (12-month) iRPE cells present various on cell size, more complexity of the cytoplasm, and more cell debris in the entire population. Right panel images represent the gating of GFP positive iRPE cells, which reflects the transduction rate of AAV2. 12-month cultured iRPE have lower AAV transduction rate than 6-month cultured iRPE cells. SSC, side scatter (reflecting complexity); FSC, forward scatter (reflecting size). (**B**) Quantification result of AAV2 and AAV5 transduction rate of 6-month culture iRPE and 12-month cultured iRPE, respectively. Data presented as Average ± SD, significance is calculated by t-test, n=4, P*<0.05, P**<0.01. (**C**) Trending of AAV2 and AAV5 transduction rate changing according to culturing time increasing, respectively. (**D**) Changing of transduction rate according to culturing time is not altering the personalized AAV serotype preference. Data presented as mean ± SD, significance is calculated by t-test, n=4, P>0.05.

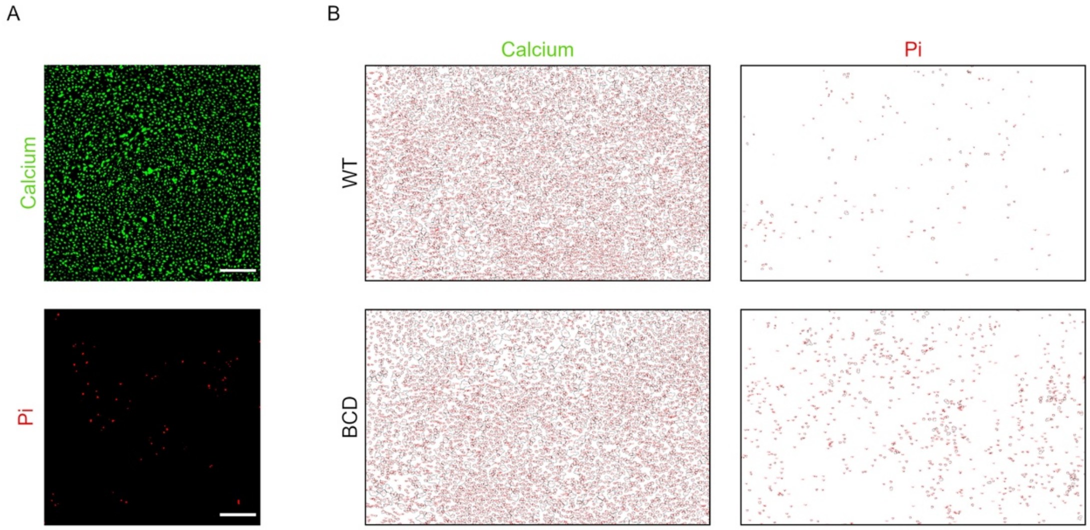

Supplemental Figure 7. Images of processing cell viability quantification. (**A**). Upper picture: live iRPE cells labeled by Calcein AM present green fluorescent signal; Lower picture: dead iRPE cells labeled by Propidium Iodide (PI) present red fluorescent signal. Scale bar: 20um. (**B**). Binary images (converted from fluorescent images) with counted particles of iRPE from WT and BCD patient after blue light exposure. Sample names labeled within each image. Scale bar: 50um

Supplemental Table 1. AAV-*CYP4V2* vector list.

**Serotype**	**Promoter**	**Transgene Packaged**	**Other regulatory elements**	**Manufacturer**
**AAV2**	CAG	human *CYP4V2* cDNA (codon-optimized)	WPRE enhancer, bGH PolyA	Vector Biolabs
**AAV5**	CAG	human *CYP4V2* cDNA	WPRE enhancer, bGH PolyA	Vector Biolabs
**AAV5-op**	CAG	human *CYP4V2* cDNA (codon-optimized)	WPRE enhancer, bGH PolyA	Vector Biolabs
**scAAV1***	EFS	human *CYP4V2* cDNA (codon-optimized)	Small PolyA (SPA)	Vector Biolabs
**scAAV5***	EFS	human *CYP4V2* cDNA (codon-optimized)	Small PolyA (SPA)	Vector Biolabs
**scAAV9***	EFS	human *CYP4V2* cDNA (codon-optimized)	Small PolyA (SPA)	Vector Biolabs
**AAV2**	CAG	human *CYP4V2* cDNA	WPRE enhancer, bGH PolyA	Andelyn Biosciences
**AAV5**	CAG	human *CYP4V2* cDNA	WPRE enhancer, bGH PolyA	Andelyn Biosciences

* “sc” indicates self-complementary.

Supplemental Table 2. Therapeutic outcomes of Individual BCD patient cell-based model from different AAV-CYP4V2 treatment strategies.

Outcomes	Samples	Treatment				
		No treatment	AAV2 high dosage	AAV2 low dosage	AAV5 high dosage	AAV5 low dosage
ROS reading	BCD-P1	103683	81566	81193	77522	93817
	BCD-P2	84990	72474	71814	66016	68739
	BCD-P3	81068	55349	59164	57922	63862
	BCD-P4	107352	80756	98409	87142	102033
	BCD-P5	95155	75942	81437	71174	80479
	BCD-P6	99182	62628	81688	70541	99111
4-HNE ug/ml	BCD-P1	116.25	54.98	64.40	44.31	83.83
	BCD-P2	76.88	55.91	67.45	71.36	73.03
	BCD-P3	65.19	45.65	52.32	62.19	63.99
	BCD-P4	119.51	68.69	89.82	62.38	90.85
	BCD-P5	118.89	88.02	91.69	74.96	99.87
	BCD-P6	104.38	68.50	77.41	65.91	74.47
Cell death%	BCD-P1	23.28%	7.55%	11.16%	8.13%	20.33%
	BCD-P2	17.24%	10.78%	11.85%	8.07%	12.77%
	BCD-P3	15.65%	8.51%	13.42%	10.95%	15.65%
	BCD-P4	25.56%	14.70%	28.27%	15.50%	26.73%
	BCD-P5	20.47%	13.81%	20.70%	13.24%	18.77%
	BCD-P6	24.14%	11.57%	21.99%	16.50%	24.40%

Supplemental Table 3. eGFP positive rate of individual iRPE subjects after AAV-eGFP transduction.

Subject / Serotype	BCD-P1	BCD-P2	BCD-P3	BCD-P4	BCD-P5	BCD-P6	WT1	WT2	WT3
**AAV2**	13.66%	22.91%	11.68%	20.10%	46.07%	32.39%	45.71%	50.22%	4.67%
**AAV5**	25.04%	57.16%	25.43%	8.24%	60.45%	22.61%	77.59%	76.84%	29.55%

Supplemental Table 4. Material information used in CRISPR/CAS9 mediated gene editing in BCD-P1 iPSCs

**gRNA sequence**	UUCAUUGGCGUUCAUUUCAU
**Single strand donor template sequence**	TAGCATATTTTATAAGAAAATGTGTTAACTAGGGTGCATCCAAGTCCAAACAGAAGCAT GTGATTATCATTCAAATCATACAGGTCATCGCTGAACGGGCtAATGAAATGAACGCtAAT GAAGACTGTAGAGGTGATGGCAGGGGCTCTGCCCCCTCCAAAAATAAACGCAGGGCC TTTCTTGACTTGCTTTTAAGTGT
**Primer of amplifying the CYP4V2 HDR**	Forward: AGAGCCTATGTTGTCGAAATGTTG<br>Reverse: GCCTGTTCCCTTCGTCATCA
