# Supplementary material for: Evaluating precision medicine approaches for gene therapy in patient-specific cellular models of Bietti crystalline dystrophy
Source: JCI Insight. 2024 Jul 16;9(16):e177231. doi: 10.1172/jci.insight.177231 (PMC11343589; doi:10.1172/jci.insight.177231)

Figure 1B BCD-P1_P6 CYP4V2

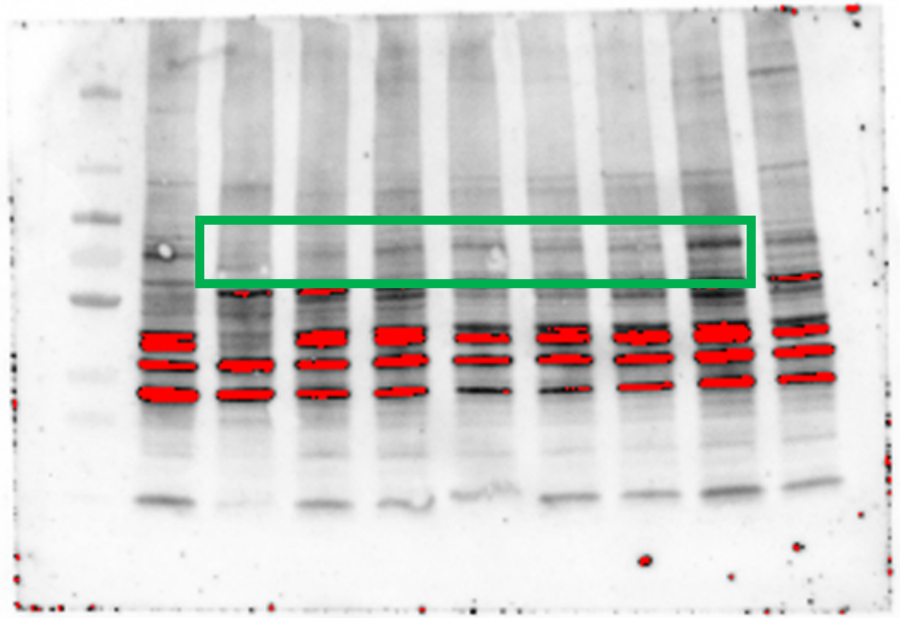

GAPDH

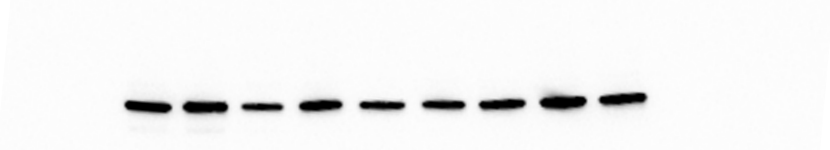

Full unedited gel for Figure S3B

CYP4V2

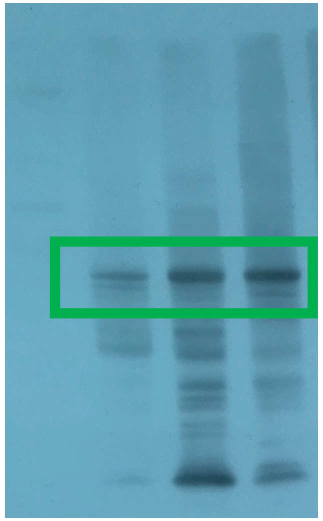

GAPDH

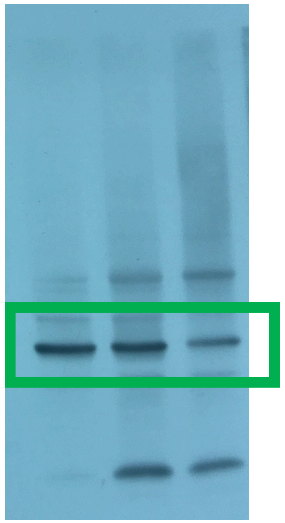

RPE65

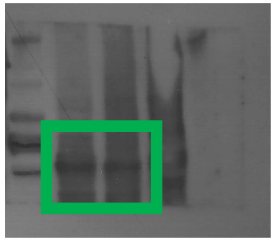

CRALBP

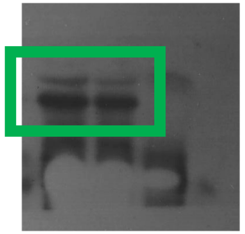

Full unedited gel for Figure S1D: P1-P3

RPE65

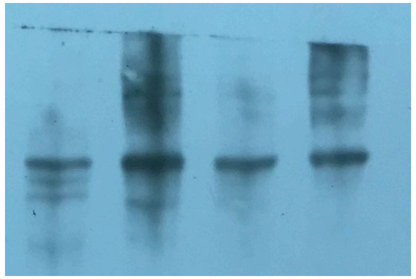

GAPDH

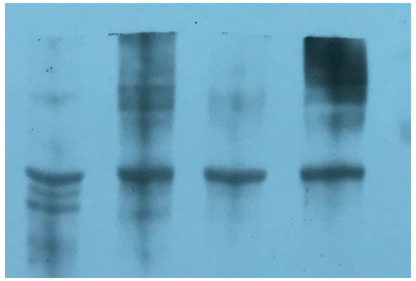

CRALBP

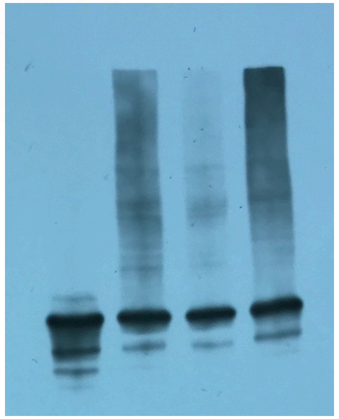

GAPDH

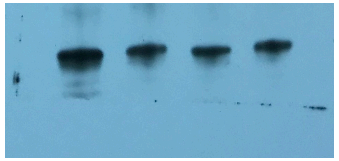

Full unedited gel for Figure S1D: P4-P6

GAPDH

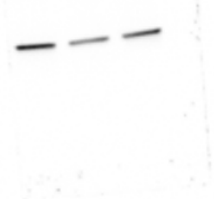

CRALBP

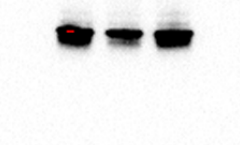

RPE65

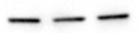

GAPDH

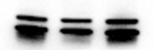

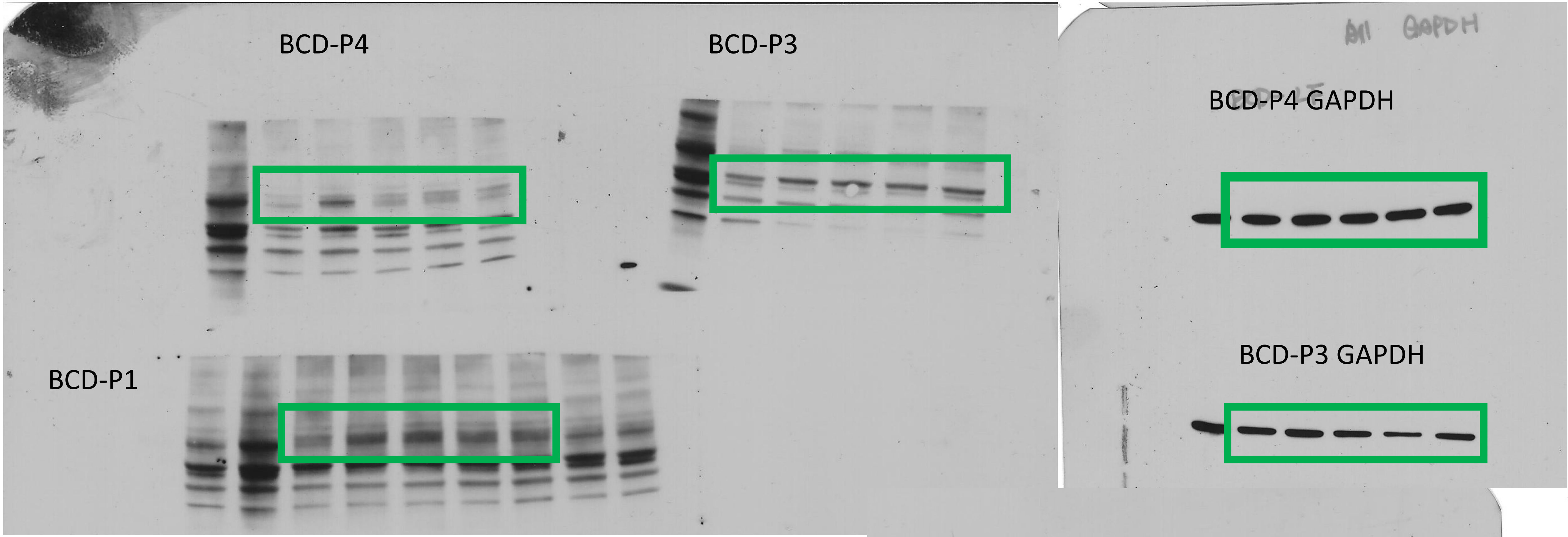

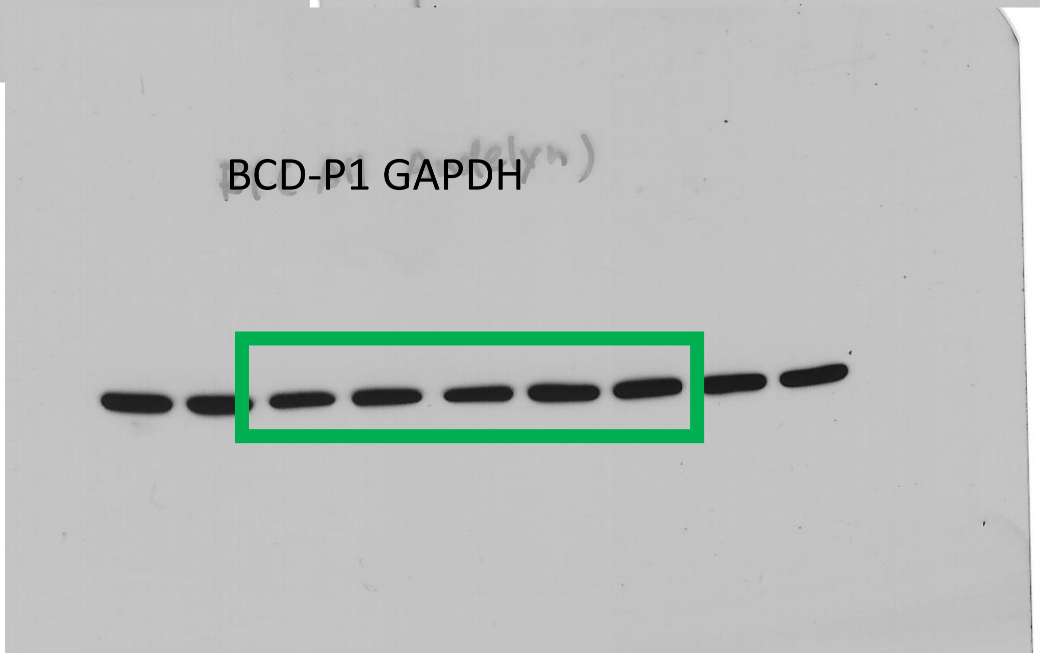

Figure 4A, BCD-P1, P3, P4

Figure 4A, BCD-P2, P5, P6

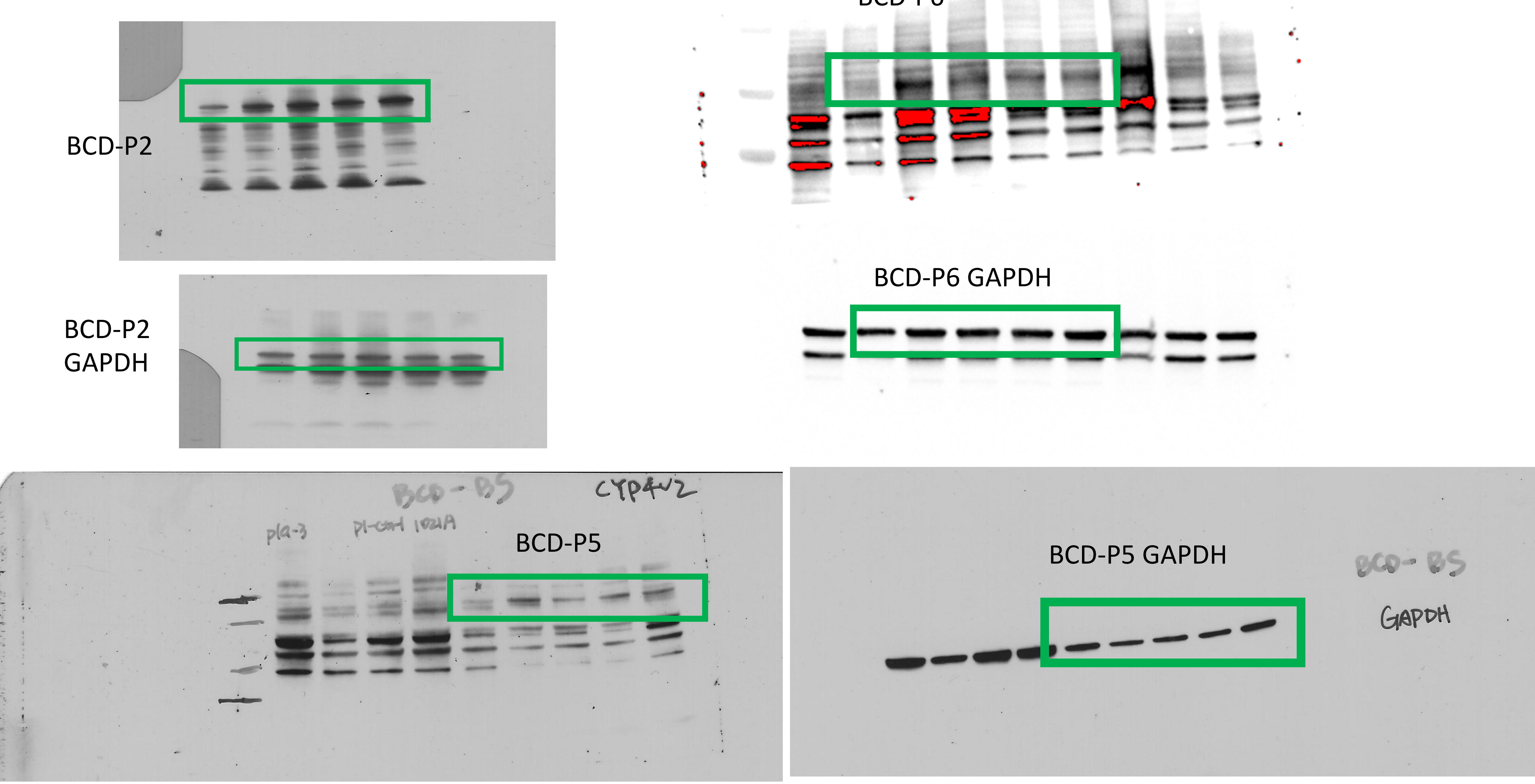

Full unedited gel for Figure S1E

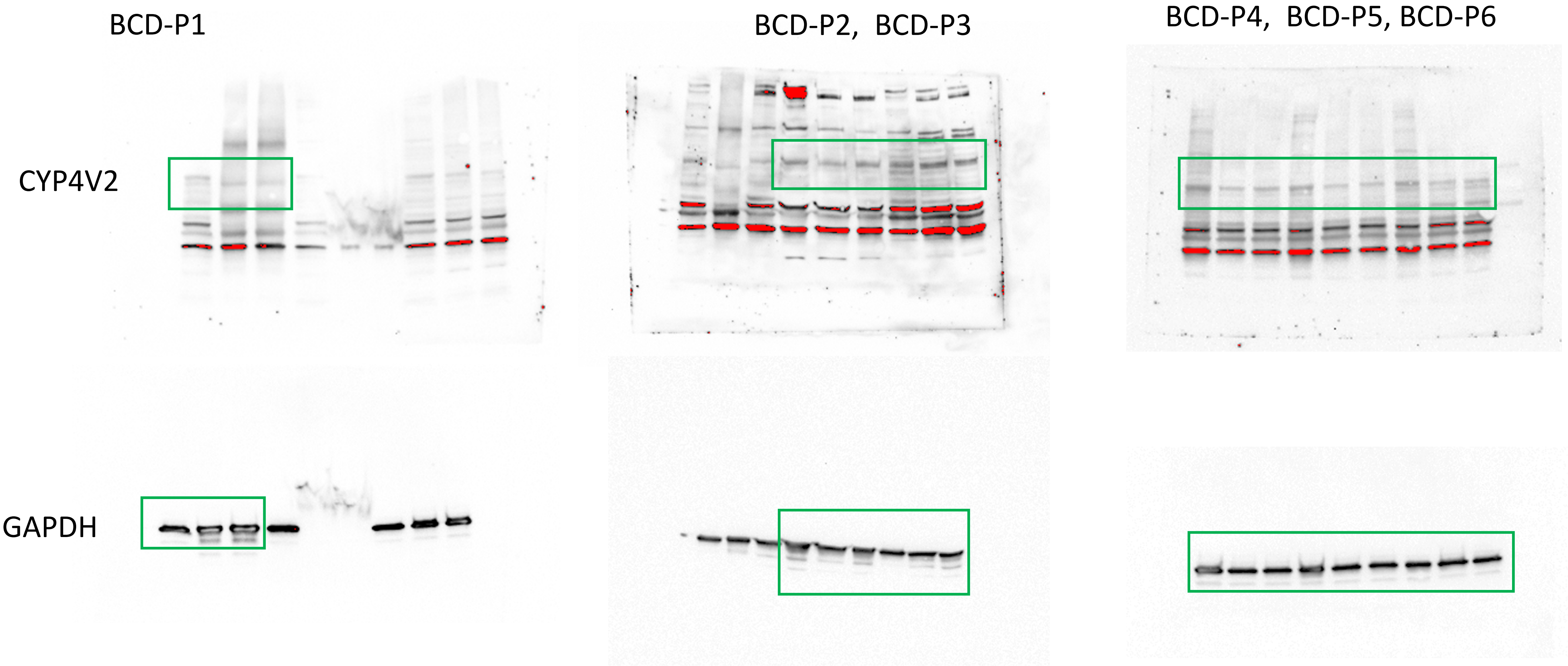

Supplement: Unedited blot and gel images [file jciinsight-9-177231-s242.pdf]
